# Changes in seminal plasma microecological dynamics and the mechanistic impact of core metabolite hexadecanamide in asthenozoospermia patients

**DOI:** 10.1002/imt2.166

**Published:** 2024-01-25

**Authors:** Baoquan Han, Yongyong Wang, Wei Ge, Junjie Wang, Shuai Yu, Jiamao Yan, Lei Hua, Xiaoyuan Zhang, Zihui Yan, Lu Wang, Jinxin Zhao, Cong Huang, Bo Yang, Yan Wang, Qian Ma, Yong Zhao, Hui Jiang, Yunqi Zhang, Shaolin Liang, Jianjuan Zhao, Zhongyi Sun, Wei Shen, Yaoting Gui

**Affiliations:** ^1^ Department of Urology Shenzhen University General Hospital Shenzhen China; ^2^ Shenzhen Key Laboratory of Male Reproductive Medicine and Genetics, Institute of Urology, Peking University Shenzhen Hospital Shenzhen‐Peking University‐The Hong Kong University of Science and Technology Medical Center Shenzhen China; ^3^ Department of Reproductive Medicine, Qingdao Hospital University of Healthy and Rehabilitation Sciences (Qingdao Municipal Hospital) Qingdao China; ^4^ College of Life Sciences Qingdao Agricultural University Qingdao China; ^5^ Department of Dermatology, Skin Research Institute of Peking University Shenzhen Hospital, Peking University Shenzhen Hospital Shenzhen Peking University‐The Hong Kong University of Science and Technology Medical Center Shenzhen China; ^6^ Department of Urology Peking University Shenzhen Hospital Shenzhen China; ^7^ State Key Laboratory of Animal Nutrition, Institute of Animal Sciences Chinese Academy of Agricultural Sciences Beijing China; ^8^ STI‐Zhilian Research Institute for Innovation and Digital Health Beijing China; ^9^ Institute for Six‐sector Economy Fudan University Shanghai China

**Keywords:** 16s rDNA sequencing, asthenozoospermia, hexadecanamide, multi‐omics analysis, seminal plasma metabolome, seminal plasma microbiota, sperm motility

## Abstract

Asthenozoospermia (AZS) is a prevalent contributor to male infertility, characterized by a substantial decline in sperm motility. In recent years, large‐scale studies have explored the interplay between the male reproductive system's microecology and its implications for reproductive health. Nevertheless, the direct association between seminal microecology and male infertility pathogenesis remains inconclusive. This study used 16S rDNA sequencing and multi‐omics analysis to conduct a comprehensive investigation of the seminal microbial community and metabolites in AZS patients. Patients were categorized into four distinct groups: Normal, mild AZS (AZS‐I), moderate AZS (AZS‐II), and severe AZS (AZS‐III). Microbiome differential abundance analysis revealed significant differences in microbial composition and metabolite profiles within the seminal plasma of these groups. Subsequently, patients were classified into a control group (Normal and AZS‐I) and an AZS group (AZS‐II and AZS‐III). Correlation and cross‐reference analyses identified distinct microbial genera and metabolites. Notably, the AZS group exhibited a reduced abundance of bacterial genera such as *Pseudomonas, Serratia*, and *Methylobacterium‐Methylorubrum* in seminal plasma, positively correlating with core differential metabolite (hexadecanamide). Conversely, the AZS group displayed an increased abundance of bacterial genera such as *Uruburuella, Vibrio*, and *Pseudoalteromonas*, with a negative correlation with core differential metabolite (hexadecanamide). In vitro and in vivo experiments validated that hexadecanamide significantly enhanced sperm motility. Using predictive metabolite‐targeting gene analysis and single‐cell transcriptome sequencing, we profiled the gene expression of candidate target genes *PAOX* and *CA2*. Protein immunoblotting techniques validated the upregulation protein levels of PAOX and CA2 in sperm samples after hexadecanamide treatment, enhancing sperm motility. In conclusion, this study uncovered a significant correlation between six microbial genera in seminal plasma and the content of the metabolite hexadecanamide, which is related to AZS. Hexadecanamide notably enhances sperm motility, suggesting its potential integration into clinical strategies for managing AZS, providing a foundational framework for diagnostic and therapeutic advancements.

## Highlights

- This study substantiates the potential clinical diagnostic utility of microbial composition and specific metabolites within seminal plasma for the asthenozoospermia (AZS) identification.
- Divergent microbial compositions and metabolite profiles within seminal plasma are discernible across varying degrees of AZS severity.
- Validation via in vitro and in vivo experiments corroborates hexadecanamide as a crucial metabolite influencing sperm motility in AZS, coupled with the dysregulation of target proteins (PAOX and CA2).

## Graphical abstract

In the onset of asthenozoospermia (AZS), the presence of six genera of bacteria (*Pseudomonas, Serratia, Methylobacterium‐Methylorubrum, Uruburuella, Vibrio*, and *Pseudoalteromonas*) in the seminal plasma potentially induces dynamic changes that ultimately diminish the synthesis of hexadecanamide. This reduction in hexadecanamide content in seminal plasma contributes to a subsequent decline in sperm motility. This reduced motility can be attributed to the downregulation of the levels of two key proteins, PAOX and CA2, within sperm cells.

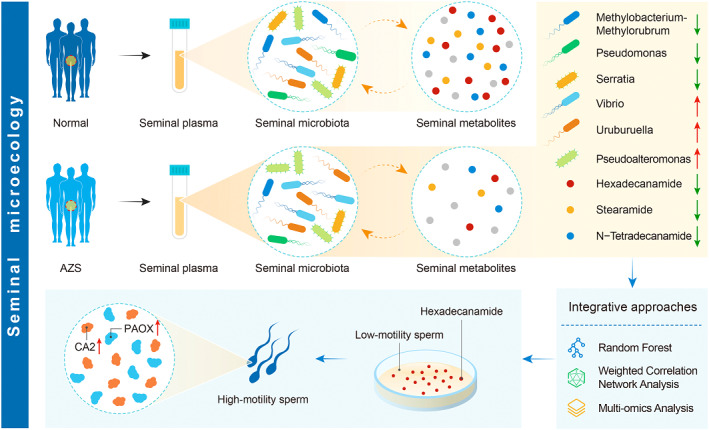

## INTRODUCTION

Although the field of microbiome has a substantial influence on the realm of biology, the microbial compositions inhabiting the male and female reproductive systems are not yet comprehensively understood. Emerging empirical evidence suggests that the male seminal microbiota is considered a key factor influencing the reproductive health of couples, pregnancy outcomes, and the well‐being of offspring [1]. Nevertheless, only a restricted number of studies have delved comprehensively into the composition and functionality of seminal microecology and its association with the development of male infertility [2]. From a worldwide perspective, infertility has surged to a prevalence rate of 15%, and currently ranks as the third most prevalent condition, following cancer and cardiovascular disorders. Approximately half of infertility cases are ascribed to male‐related factors [3]. Asthenozoospermia (AZS) is a prevalent etiological factor contributing to male infertility, characterized by diminished sperm motility [4]. According to the guidelines promulgated by the World Health Organization in 2010, AZS patients are clinically delineated as individuals exhibiting total sperm sample motility of <40% and progressive motility lower than 32%. The etiology of AZS is multifaceted, influenced by various determinants including endocrine disruptors, environmental stressors, individual life trajectories, and the process of aging, although the exact cause remains uncertain.

Seminal plasma constitutes over 90% of the human ejaculate volume, primarily composed of secretions from various anatomical structures, including the urethral bulb glands, prostate, periurethral glands, epididymis, and seminal vesicles [5]. Recent studies employing sequencing technologies have revealed the existence of a distinct microbial community in semen [6, 7]. However, the precise role of microorganisms in semen remains unclear. Notably, multiple microbiota studies have substantiated the presence of diverse microbial communities in semen, with specific microorganisms potentially influencing sperm quality. For instance, Hou et al. [6] observed no pronounced disparities in the distribution of bacterial communities in the seminal microbiota of healthy and infertile men. Nevertheless, the abundant of *Anaerococcus* in the seminal microbiota may be associated with male infertility. A recent study identified numerous bacterial species in the semen of AZS patients, and predictive models suggested that specific bacterial species, such as *Ureaplasma, Bacillus*, and *Anaerobes*, may detrimentally affect sperm quality [8]. In 2021, Lundy et al. [2] reported a notable increase in semen α‐diversity and apparent β‐diversity, along with a substantial rise in the abundance of *Aerococcus* in the semen of infertile men. Moreover, *Pseudomonas* abundance displayed a negative correlation with sperm concentration, while it exhibited a direct correlation with the total count of motile sperm [2]. In summary, there is growing evidence indicating a potential connection between microorganisms and sperm parameters in semen, which may be related to male infertility. However, it is essential to acknowledge that the results from various existing semen microbiome studies are inconsistent, highlighting the necessity for additional research to definitively establish the relationship between male infertility and semen microbiota.

In recent years, numerous studies have conducted investigations into the impact of seminal plasma health on sperm motility, with a specific focus on the comprehensive analysis of the metabolic profiles within seminal plasma such as nuclear magnetic resonance by Jayaraman et al. [9] and liquid chromatography mass spectrometry (LC‐MS) by Chen et al. [10]. Through the analysis with LC‐MS analysis, potential biomarkers in the seminal plasma from individuals with weak spermatozoa were primarily concentrated on metabolites related to oxidative stress. It is noteworthy that prior research has validated the association between the oxidative stress response and anomalies in spermatogenesis [11], therefore, emphasizing the potential for using metabolomics for unraveling the pathogenesis of male infertility. Moreover, a metabolomics study conducted in 2014 enhanced our comprehension of potential biomarkers for the diagnosis and treatment of male infertility [12]. Significantly, this investigation reaffirmed the crucial functions of l‐carnitine and acetyl l‐carnitine in sperm metabolism and maturation. Subsequently, in 2020, Xu et al. [13] meticulously characterized the composition of semen metabolites in patients afflicted by weak sperm vitality in comparison to a control group comprising healthy individuals. Concurrently, Li et al. [14] identified nine principal components within the distinctive metabolite composition of semen from individuals with compromised sperm vitality. These components encompassed creatinine, uric acid, N6‐methyladenine, uridine, taurine, carnitine, nicotinamide, N‐acetylaminoacetic acid, and l‐palmitoylcarnitine. Although these research findings marked initial evidence into understanding changes in the metabolite composition of seminal plasma in patients with AZS, it is imperative to acknowledge that these results exhibited a degree of uniformity and lack of precision. These limitations were often attributed to the absence of accurate and detailed patient subgroups. Consequently, they were insufficient in establishing a conclusive relationship between alterations in seminal plasma metabolite composition and the incidence of AZS.

To date, there exists a shortage of comprehensive and meticulously detailed analyses pertaining to the microecological dynamics of seminal plasma in individuals afflicted with AZS. This dearth includes both the seminal microbiota and metabolite composition. The objective of this study is to delineate the composition of the seminal plasma microbial community and the metabolic profile, with a particular focus on potential associations with the occurrence of AZS. It is our aspiration to conduct a precise and rigorous examination of the intricate relationship between variations in seminal plasma microecological dynamics and the pathogenesis of AZS. Furthermore, this research strives to identify key microorganisms or fundamental metabolites that could serve as reliable candidates for screening in the clinical management of AZS. The ultimate goal is to furnish a robust theoretical framework for the advancement of diagnosis and treatment strategies for AZS.

## RESULTS

### Patient baseline characteristics

**Figure 1:**
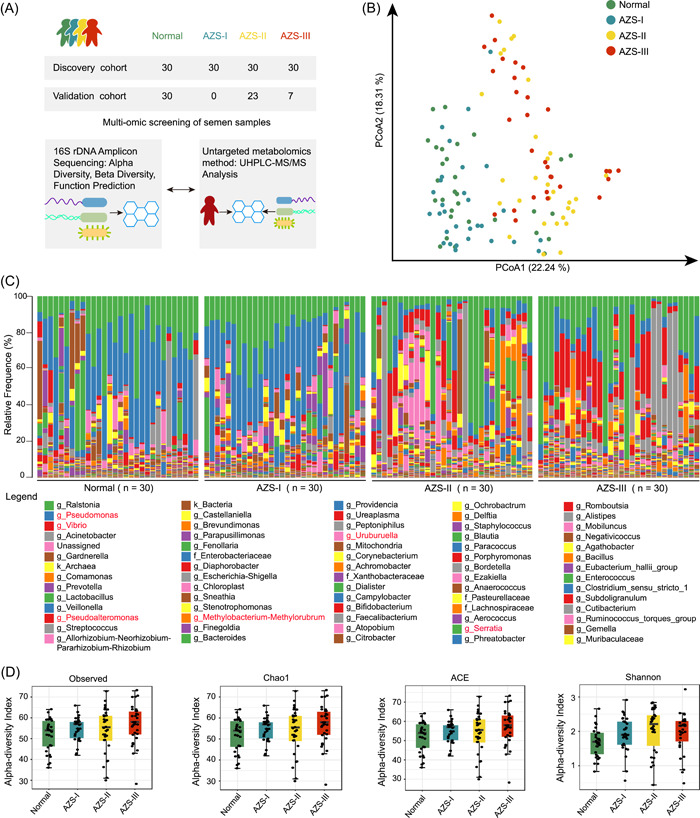
Study design and analysis of microbiota community structure changes associated with asthenozoospermia (AZS). (A) Overall study design. Mild AZS: AZS‐I; moderate AZS: AZS‐II; severe AZS: AZS‐III. (B) β‐diversity analysis using principal coordinates analysis (PCoA). (C) Species composition and abundance presentation: the legend provides information regarding the representation of relative frequency (%) of species within each community. (D) α‐diversity analysis across diverse groups: (1) Observed: counts the number of unique species observed in a given community. (2) Chao1: estimator used to estimate the true species richness within a single community. (3) Abundance‐based coverage estimator (ACE): estimator used to assess species richness within a community. (4) Shannon: measures the diversity within a community, considering both species' richness and evenness.

A total of 120 patients participated in the study, with 30 individuals in each of the four groups: Normal, mild AZS (AZS‐I), moderate AZS (AZS‐II), and severe AZS (AZS‐III) (Figure 1A and Table S1). The information associated with the grouping of patients can be found within Table S2. Notably, there were no significant disparities in terms of age and body mass index across the four groups. However, statistically significant variations were observed in various semen parameters, encompassing semen volume, progressive motility, non‐progressive motility, immotility, sperm concentration, and normal morphology. An overview of the baseline clinical characteristics can be found in Table S3.

### Analysis of microbial community structure changes and species differences in AZS

Figure 1B,C illustrate a richly diverse microbial community. Within the realm of microbial communities, there is a distinct observation: the Normal group and the AZS‐I group manifest akin community structures. Similarly, the AZS‐II and AZS‐III groups exhibit comparable community structures. Further examination in Figure 1C reveals that AZS‐II and AZS‐III, in contrast to the Normal and AZS‐I groups, present notable variations in the composition of several genera, notably *Pseudomonas, Serratia, Pseudoalteromonas, Uruburuella, Vibrio*, and *Methylobacterium Methylorubrum*. Subsequent to the α‐diversity analysis (Figure 1D), it is discerned that the AZS‐II and AZS‐III groups display higher values across all four indices (abundance‐based coverage estimator, Chao1, Observed, and Shannon) in comparison with the other two groups. This observation indicates the heightened species diversity within the moderate‐to‐severe AZS groups. Notably, it establishes a significant correlation between the richness and diversity of species in seminal samples and the manifestation of moderate‐to‐severe AZS. Furthermore, β‐diversity analysis, executed through principal coordinates analysis (PCoA), unveils distinctions between groups, as depicted in Figure 1B. Specifically, it has become evident that the microbial community composition in the Normal and AZS‐I groups demonstrates a greater degree of similarity, while the AZS‐II and AZS‐III groups exhibit a closer resemblance. These findings serve as vital benchmarks for the subsequent phases of our analysis.

Subsequently, we meticulously selected the top 30 species at each taxonomic level (phylum, class, order, family, genus) for individual samples or subgroups, guided by the species annotation results. Cumulative bar plots were generated, facilitating the visualization of species with notable relative abundances at various taxonomic tiers for each sample, indicating their proportions across these levels. As illustrated in Figure S1A, a significant reduction in the relative abundance of *Pseudomonas* in AZS‐II and AZS‐III was observed when compared to Normal and AZS‐I samples. Conversely, the relative abundance of *Vibrio* and *Pseudoalteromonas* exhibited a significant increase. These analyses offer preliminary insights into the correlation between the abundance of distinct genera in seminal samples and the emergence of moderate‐to‐severe AZS. Furthermore, we conducted a thorough examination of species annotation results, focusing on common and unique amplicon sequence variants (ASVs) among different comparative groups, adhering to denoising protocols and research requisites. The outcomes illustrated in Figure S1B, reveal that AZS‐II and AZS‐III cohorts share 954 ASVs (absent in Normal and AZS‐I), with AZS‐II accounting for 1917 unique ASVs and AZS‐III encompassing 1686 unique ASVs. In contrast, Normal and AZS‐I exhibit 693 ASVs (absent in AZS‐II and AZS‐III), with Normal featuring 2376 unique ASVs and AZS‐I comprising 1235 unique ASVs. This observation strongly indicates a distinctive composition of seminal microbiota in populations afflicted with moderate‐to‐severe AZS, setting them apart from individuals with normal and mild AZS conditions.

**Figure 2:**
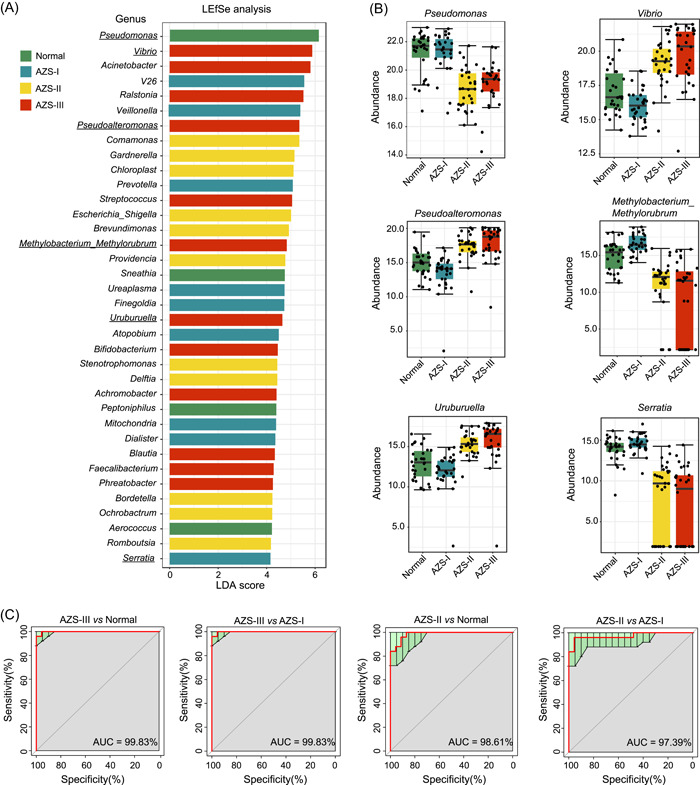
Linear discriminant analysis effect size (LEfSe) and Random Forest analyses of microbiota community. (A) LEfSe analysis of microbiota community between groups with linear discriminant analysis (LDA) scores. (B) Distribution of microbial abundance for the six genera. (C) Random Forest model performance evaluation via receiver operating characteristic (ROC) curves. AUC, area under the ROC curve; AZS, asthenozoospermia; AZS‐I, mild AZS; AZS‐II, moderate AZS; AZS‐III, severe AZS.

Expanding upon this observation, we conducted a differential analysis of the disparities within microbial communities present in seminal samples from individuals with Normal, AZS‐I, AZS‐II, and AZS‐III conditions, as depicted in Figure S1C. In contrast to the Normal group, AZS‐I, AZS‐II, and AZS‐III exhibited variations in the composition of different genera, resulting in 61, 88, and 145 distinct genera, respectively. Within these comparisons, 5, 29, and 86 genera were upregulated, whereas 56, 59, and 59 genera were downregulated in AZS‐I, AZS‐II, and AZS‐III, respectively. In addition, when compared to AZS‐I, AZS‐II and AZS‐III showed unique microbial profiles comprising 45 and 100 distinct genera, respectively. Among these, 34 and 84 genera were upregulated, while 11 and 16 genera were downregulated in AZS‐II and AZS‐III, respectively. These findings indicate substantial variations in seminal microbiota among AZS patients, with the most notable differences occurring in individuals with moderate‐to‐severe AZS. Furthermore, we performed cross‐referenced comparisons involving AZS‐II and Normal, AZS‐III and Normal, AZS‐II and AZS‐I, and AZS‐III and AZS‐I, serving as four control groups. This rigorous analysis identified 17 genera closely linked to the manifestation of moderate‐to‐severe AZS, including *Pseudomonas, Serratia, Methylobacterium‐Methylorubrum, Uruburuella, Vibrio*, and *Pseudomonas* (Figure S1D). These findings further support the potential role of these six genera in the development of moderate‐to‐severe AZS. In addition, species differential analysis was executed using linear discriminant analysis effect size (Figure 2A) and statistical analysis of metagenomic profiles (STAMP) analysis (Figure S2A). Figure 2B provides a box plot that visually represents the distribution of microbial abundance among the six genera within four distinct patient groups. These methods confirmed the significant upregulation or downregulation of these six genera in patients afflicted with moderate‐to‐severe AZS within the four comparative groups, providing additional evidence of their close association with the occurrence of moderate‐to‐severe AZS.

Subsequently, to investigate species exhibiting noteworthy distinctions between groups, we executed a hypothesis testing procedure at the species level, evaluating species abundance using the *t* test method on the species abundance data derived from various strata. This statistical examination yielded *p*‐values, permitting the identification of species with substantial intergroup differences. Furthermore, we generated plots illustrating the distribution of these species' abundance across different groups. The results illustrated in Figure S2B and S3A–C revealed significant variations in the abundance of the six aforementioned differential genera when comparing AZS‐II or AZS‐III with the Normal or AZS‐I groups. These findings provide further support for the putative significance of these genera in the context of moderate‐to‐severe AZS.

### Correlation between seminal microbiota functions and microbial environmental factors in AZS patients

**Figure 3:**
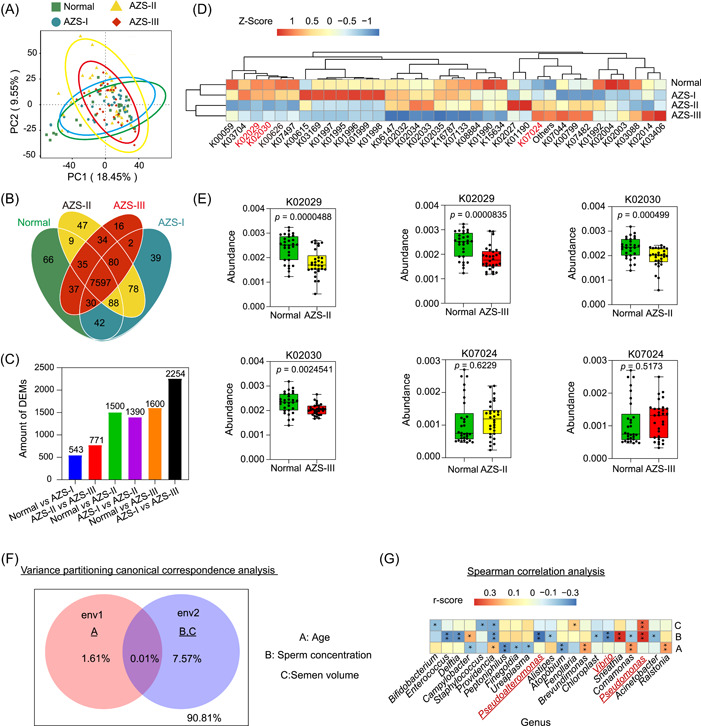
Functional prediction of seminal plasma microbiota and environmental correlation analysis in diverse asthenozoospermia (AZS) patients. (A) Principal component analysis (PCA) plot: visualizing sample distribution based on functional prediction analysis. (B) Venn diagrams to analyze common and unique metabolic pathways in different groups. (C) Statistical analysis of differential metabolites from different comparative groups involves the compilation and assessment of data related to metabolites that exhibit significant differences in abundance between these groups. (D) Heatmap display of 16S sequencing data after corresponding functional prediction. (E) Metabolic function display of three metabolic pathways in different groups. (F) Variance partitioning canonical correspondence analysis between microbiota distribution, age, sperm concentration, and semen volume. (G) Spearman correlation analysis between microbiota distribution, age, sperm concentration, and semen volume.

Following the elucidation of microbiota community structure and species variations across different samples, we employed PICRUSt2 to predict functional attributes using the Kyoto Encyclopedia of Genes and Genomes (KEGG) database. Initially, we conducted a clustering analysis of the relative abundance of functional annotations. Figure 3A–C show the outcomes of principal component analysis, the distribution pattern of differential metabolites (DEMs), and a Venn diagram demonstrating the intersection of KEGG pathways across distinct groups. As depicted in Figure 3D,E, functional attributes represented by K02029, K02030, and K07024 displayed similar decreasing or increasing trends in AZS‐II and AZS‐III patients, whereas corresponding increasing or decreasing trends were observed in the Normal and AZS‐I groups. Notably, K02029 and K02030 are associated with amino acid transport functions, implying a potential connection between sperm viability and amino acid transport functions. Additionally, K07024 is correlated with sucrose phosphatase function; however, the precise functional mechanisms require further confirmation and investigation.

Expanding upon the previous analyses, we delved into an environmental factor correlation study using Spearman correlation and variance partitioning analysis (VPA). This allowed us to investigate the relationships between age, sperm concentration, semen volume, and the composition of the seminal microbiota in the four groups. The outcomes of the VPA, illustrated in Figure 3F, highlighted that sperm concentration and semen volume exert a substantial explanatory influence on the distribution of species within the seminal microbiota. This underscores the potential significance of the seminal microbiota in influencing semen quality. As depicted in Figure 3G, *Pseudoalteromonas* and *Vibrio* exhibited a negative correlation with sperm concentration, whereas *Pseudomonas* displayed positive correlations with sperm concentration. *Pseudomonas* also exhibited a positive correlation with semen volume. These findings provide additional support for the intimate connection between seminal microbiota and semen quality, although the precise underlying mechanisms warrant further investigation.

### Significant association of AZS with seminal metabolic profiles

**Figure 4:**
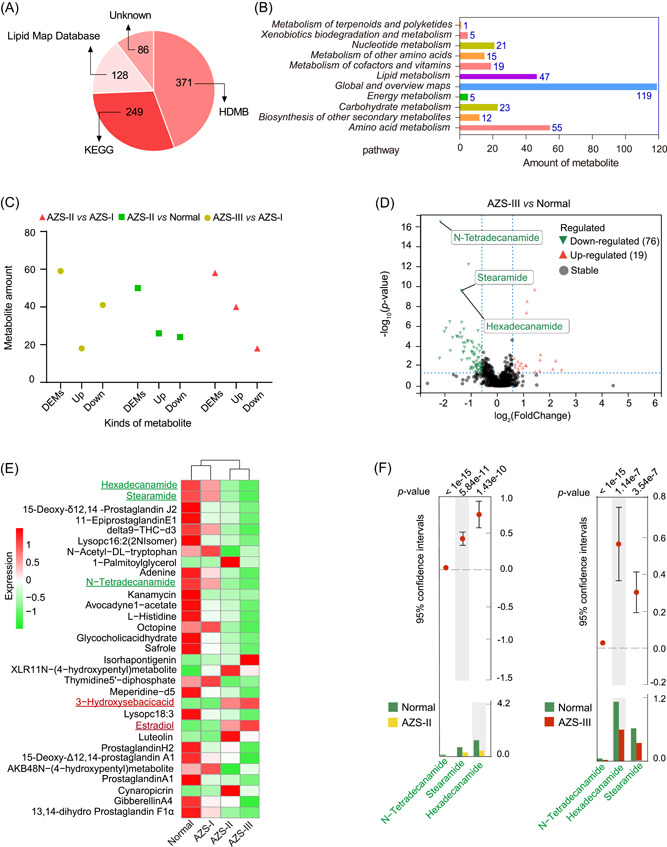
Asthenozoospermia (AZS)‐associated broad changes in seminal plasma metabolic profile. (A) Quantitative distribution of metabolites based on different databases. (B) Secondary metabolic pathway distribution of metabolites based on the Kyoto Encyclopedia of Genes and Genomes (KEGG) database. (C) The statistical analysis of differential metabolites (DEMs) from diverse comparison groups. (D) The volcano map of DEMs from AZS‐III vs Normal comparison group. (E) A heatmap of common DEMs from diverse patient groups. (F) A statistical analysis of metagenomic profiles (STAMP) analysis of three metabolite profiles in diverse groups. AZS‐I, mild AZS; AZS‐II, moderate AZS; AZS‐III, severe AZS.

We conducted an extensive analysis of the metabolomic profiles in seminal samples from different groups. As demonstrated in Figure 4A and detailed in Table S4, a total of 834 metabolites were successfully annotated by comparing them with reference data derived from the KEGG database. Notably, among these, 249 features (constituting 29.86% of the total) were identified. Among these identified pathways, the predominant metabolic pathways included lipid metabolism and amino acid metabolism (Figure 4B). Additionally, by cross‐referencing the data with the Human Metabolome Database (HMDB), 371 metabolites (comprising 44.48% of the total metabolites) were categorized into putative molecular classes. The top three classes primarily encompassed lipids and lipophilic molecules, organic acids and derivatives, as well as organic heterocyclic compounds (Figure S4A). Furthermore, by contrasting the data against the free resource Lipid Maps Database, 128 features (15.34%) were identified. Notably, the dominant categories within this subset included phosphatidic acids, glycerophosphoethanolamines (GP02), fatty acids and their conjugates (FA01), and glycerophosphocholines (GP01) (Figure S4B).

Contrary to the results of microbial community analysis, the PCoA suggested that differences in the distribution of the metabolome among the four groups were relatively modest (Figure S4C). Nevertheless, an analysis of similarities (Anosim) of DEMs in seminal samples indicated significant differences between groups, indicating the importance of the grouping analysis in this study (Figure S4D). Subsequent partial least squares discriminant analysis (PLS‐DA) (Figure S5A) revealed that, compared to the AZS‐III group, the spatial distribution of the Normal and AZS‐I groups displayed similarities, as did the Normal and AZS‐I groups in comparison to the AZS‐II group. This indicates that the metabolic presence patterns of the Normal and AZS‐I groups exhibited greater similarities. After performing PLS‐DA analysis and Anosim analysis of metabolites, we conducted statistical analyses of DEMs in various groups (AZS‐II vs Normal, AZS‐III vs Normal, AZS‐II vs AZS‐I, and AZS‐III vs AZS‐I), as demonstrated in Figure 4C,D and Figure S5B. In comparison with the Normal group, AZS‐II exhibited 50 DEMs, comprising 26 upregulated and 24 downregulated metabolites, whereas AZS‐III displayed 95 DEMs, including 19 upregulated and 76 downregulated. In contrast, when compared with AZS‐I, the AZS‐II group featured 58 DEMs, with 40 upregulated and 18 downregulated, and AZS‐III had 59 DEMs, comprising 18 upregulated and 41 downregulated metabolites. Furthermore, as depicted in Figure 4E, the metabolic presence patterns of the top 30 distinct metabolite features were deemed similar between the Normal and AZS‐I groups, while the AZS‐II and AZS‐III groups also exhibited closer similarities. It's noteworthy that significant differences were observed in the content of seminal metabolites between AZS‐II and AZS‐III, characterized by notably decreased levels of specific metabolites, including n‐tetradecanamide, hexadecanamide, and stearamide (referred to as core DEMs) in the seminal plasma of moderate‐to‐severe AZS patients. Conversely, 3‐hydroxysebacicacid and estradiol were significantly higher (Figure 4E). These findings elucidate significant distinctions in the content of seminal metabolites between AZS‐II and AZS‐III in contrast to Normal and AZS‐I, with a marked decrease in the levels of core DEMs in the seminal plasma of moderate‐to‐severe AZS patients. Moreover, we performed STAMP analysis of the metabolic profiles of these core DEMs in the four sample groups, revealing significant downregulation in AZS‐II and AZS‐III patients (Figure 4F). This further underscores the close association between these core DEMs and the occurrence of moderate‐to‐severe AZS. These results suggest a substantial correlation between AZS and profound alterations in the seminal metabolome, particularly concerning the occurrence of AZS‐II and AZS‐III.

**Figure 5:**
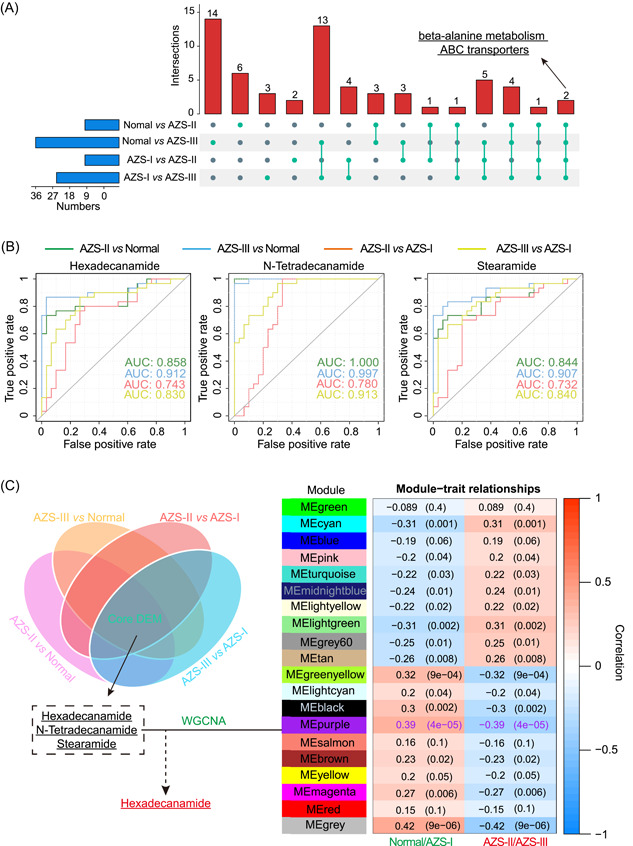
Analysis of common pathways, receiver operating characteristic (ROC Curve) analysis of core metabolites, and candidate metabolite screening. (A) Analysis of common pathways of the four comparison groups. (B) ROC curve analysis of three differential metabolites (DEMs) in diverse comparison groups. (C) Screening of the candidate metabolite combined with different methods: the left panel illustrates the selection of DEMs from various comparison groups. The right panel shows the results of weighted gene co‐expression network analysis (WGCNA) of DEMs in diverse patient groups. The middle panel highlights the determination of the candidate metabolite, hexadecanamide. AZS, asthenozoospermia; AZS‐I, mild AZS; AZS‐II, moderate AZS; AZS‐III, severe AZS.

Additionally, our correlation analysis of different metabolites across various groups (Figure S6), unveiled a notably strong positive correlation among the core DEMs, hinting at a potentially significant interplay in their synthesis. Subsequent scrutiny was directed toward the KEGG enrichment analysis of DEMs in these samples (Figure S7). By comparing these results with the pathway analysis presented in Figure 5A, our findings point to the potential pivotal roles of β‐alanine metabolism and ABC transporters in influencing sperm motility. These insights offer valuable references for our forthcoming research endeavors.

### Predicting AZS status and subtype using multi‐omics features in seminal plasma

Subsequently, we employed random forest analysis to predict the microbial and metabolic characteristics of the seminal samples within the four groups. The receiver operating characteristic (ROC) analysis results for the microbial community, as depicted in Figure 2C, revealed that both AZS‐II and AZS‐III exhibit excellent diagnostic capabilities (area under the curve [AUC] > 0.9) when compared to Normal and AZS‐I. This underscores the potential of seminal microbiota composition as a classifier for identifying moderate‐to‐severe AZS. It further signifies the clinical diagnostic utility of seminal plasma microbiota in diagnosing moderate‐to‐severe AZS, emphasizing its substantial impact on sperm motility.

Furthermore, we conducted a statistical evaluation of the ROC results for the core DEMs among different groups. The outcomes demonstrated that both AZS‐II and AZS‐III (Figure 5B) display favorable diagnostic performance (AUC > 0.7) in comparison to Normal and AZS‐I, with more robust diagnostic potential within the severe AZS group. This suggests that the distinctive levels of core DEMs in seminal plasma can serve as valuable diagnostic tools for clinical assessments of moderate‐to‐severe AZS. It also highlights the pivotal role played by these core DEMs in influencing sperm motility.

### Screening candidate metabolites via integrated‐weighted gene co‐expression network analysis (WGCNA) metabolomics analysis

Building upon the findings from PCoA analysis of seminal microbial communities and PLS‐DA analysis of seminal metabolomes within the Normal, AZS‐I, AZS‐II, and AZS‐III groups, we proceeded to conduct a secondary classification of these sample groups. Notably, the Normal and AZS‐I groups exhibited similarities akin to the control group, while the AZS‐II and AZS‐III groups were more akin to the AZS group, as depicted in Figure 5C (right panel). In this classification, we identified a total of 15 differential modules, each representing a set of metabolites closely linked to AZS. These 15 differential modules were then used to select candidate metabolites.

**Figure 6:**
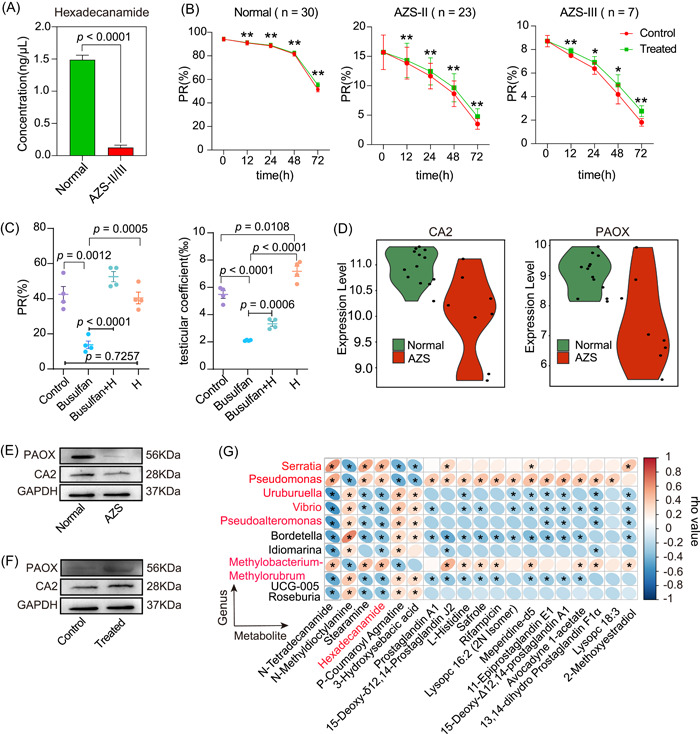
Detection of candidate metabolite levels in newly collected samples and functional verification of the candidate metabolite on sperm motility in vitro and in vivo. (A) Quantitative analysis of hexadecanamide from diverse newly collected samples. *p* < 0.05 indicates significant difference. (B) Statistical analysis of sperm motility from different groups at different time points. The asterisk (* or **) signifies a statistically significant association (*p* < 0.05 or *p* < 0.01). (C) Statistical analysis of sperm motility from different group (left panel). Statistical analysis of testicular coefficient from different groups (right panel). *p* < 0.05 indicates significant difference. (D) Expression analysis of target genes (*PAOX* and *CA2*) in sperm cells of asthenozoospermia (AZS) patients using the Gene Expression Omnibus (GEO) database. (E) Protein levels detection of PAOX and CA2 proteins in sperm samples from different patients' donors. (F) Protein levels detection of PAOX and CA2 proteins in different treatment groups from in vitro culture experiment. Control, untreated group; Treated, hexadecanamide‐treatment group. (G) AZS‐associated changes in metabolic features and their microbial associations: The asterisk (*) signifies a statistically significant association (*p* < 0.05), with the blue block indicating a negative correlation and the red block signifying a positive correlation between the variables. AZS‐I, mild AZS; AZS‐II, moderate AZS; AZS‐III, severe AZS.

In alignment with the outcomes of the differential metabolite analysis and WGCNA analysis, we initially chose the top 15 DEMs from each of the four comparative groups (AZS‐II vs Normal, AZS‐III vs Normal, AZS‐II vs AZS‐I, and AZS‐III vs AZS‐I) for further evaluation. Figure 5C (left panel) showcases the selection of three core DEMs, including n‐tetradecanamide, hexadecanamide, and stearamide. Subsequently, we cross‐referenced these core DEMs with the 15 differential modules derived from the WGCNA analysis, ultimately leading to the identification of the candidate differential metabolite‐hexadecanamide (Figure 5C). To corroborate this observation, we proceeded to perform a quantitative analysis of hexadecanamide across a range of samples, which included newly‐collected Normal samples and samples from individuals diagnosed with AZS‐II and AZS‐III. The findings, illustrated in Figure 6A, clearly exhibited a substantial reduction in hexadecanamide levels among AZS patients. This aligns with the outcomes of the metabolomics analysis, further underscoring the potential pivotal role of hexadecanamide in influencing sperm motility.

### Validating the promotional effect of hexadecanamide on sperm motility in Normal/AZS cohorts and a murine oligoasthenospermia model

To further substantiate the role of hexadecanamide in enhancing sperm motility, we initiated a validation cohort study and collected sperm samples following the same standardized protocol as previously described. The objective was to assess the stimulatory impact of hexadecanamide on sperm motility. We established the optimal concentration of hexadecanamide at 10 nM through preliminary in vitro culture experiments (Figure S8A) and subsequently conducted an extensive validation experiment. This entailed using newly‐collected sperm samples, consisting of 30 samples from the Normal group and 30 samples from the AZS group, with 23 individuals diagnosed with AZS‐II and 7 with AZS‐III. All sperm samples were treated with hexadecanamide at a concentration of 10 nM, and sperm motility rates were analyzed statistically at various time points, including 0, 12, 24, 48, and 72 h, respectively. The results, as depicted in Figure 6B, unequivocally demonstrated that hexadecanamide exerted a significant enhancing effect on sperm motility across the different groups, with a notably more substantial impact observed in the AZS‐II and AZS‐III patient cohorts. Following the completion of in vitro experiments, we proceeded to use a murine model of oligoasthenospermia induced by intraperitoneal injection of busulfan to further confirm the influence of hexadecanamide on enhancing sperm motility. Before the formal experiments, we conducted pilot experiments to determine the optimal concentration, which was established at 12.772 μg/kg (Figure S8B–D). Subsequent results from the formal experimental phase (Figure 6C) validated that the hexadecanamide treatment group exhibited a highly significant effect on testicular development and sperm motility in comparison to the control group. This outcome solidified the notion that hexadecanamide has a pronounced promotional effect on sperm motility in low‐motility sperm.

### Mechanisms of hexadecanamide‐enhanced sperm motility through CA2 and PAOX protein expression

To elucidate the mechanisms underlying the enhancement of sperm motility by hexadecanamide, we initiated a multi‐step investigation. Initially, we obtained the chemical formula of hexadecanamide from the National Center for Biotechnology Information database (https://www.ncbi.nlm.nih.gov/) and subsequently predicted its target gene set. This prediction was based on the chemical formula of hexadecanamide in conjunction with the SwissTargetPrediction database. We further refined the target gene set based on probability, ultimately identifying three potential target genes: *CA2, CA1*, and *PAOX* (Figure S8E).

Following the initial identification of these candidate target genes, we conducted an expression analysis of these genes in sperm cells of AZS patients using existing datasets from the Gene Expression Omnibus database (https://www.ncbi.nlm.nih.gov/geo/), specifically GSE6968 and GSE6872. As depicted in Figure 6D, it became evident that the expression of two genes, *CA2* and *PAOX*, was significantly downregulated in the sperm cells of AZS patients in comparison to a Normal population. Conversely, the expression of *CA1* was either not significantly different or undetected. We also performed a single‐cell resolution expression analysis of *CA2* and *PAOX* in testicular tissues using The Human Protein Atlas database in conjunction with available single‐cell transcriptome data. These results indicated that both *CA2* and *PAOX* were predominantly expressed in late or mature spermatids, with minimal expression observed in spermatocytes or spermatogonia (Figure S9). This observation further underscored the crucial roles of *CA2* and *PAOX* in the normal motility of sperm.

Building upon this groundwork, we conducted an evaluation of the protein levels of CA2 and PAOX using newly collected samples, comprising the Normal group and the AZS group. The results illustrated in Figure 6E revealed significant downregulated protein levels of CA2 and PAOX in the AZS group in comparison with the Normal group. This decline in hexadecanamide levels in the seminal plasma of AZS patients provided additional evidence that reduced hexadecanamide levels downregulated the levels of these two proteins, consequently diminishing sperm motility.

Expanding on these findings, we performed in vitro culture experiments involving the coculture of hexadecanamide with sperm for 72 h. Subsequently, we assessed the protein levels of PAOX and CA2 by collecting sperm cells from different treatment groups. The results (Figure 6F and S8F) clearly demonstrated a significant increase in the protein levels of both PAOX and CA2 in the sperm cells of the AZS group following hexadecanamide treatment. This suggested that hexadecanamide may enhance sperm motility by elevating the protein levels of the target proteins PAOX and CA2 in sperm cells.

### Metabolic feature changes associated with AZS and their microbial associations

Significantly, we employed Pearson's correlation coefficient to establish correlations between the markedly different genera and metabolites in the four comparative groups (AZS‐III vs Normal, AZS‐III vs AZS‐I, AZS‐II vs Normal, and AZS‐III vs AZS‐I). Heat maps were generated to gauge the extent of association between the diversity of species and metabolites in seminal plasma samples (Figure 6G and Figures S10 and S11). Subsequently, we conducted a Venn analysis of the differential genera derived from the four comparative groups that exhibited close associations with the core DEMs. Consequently, we identified six prominent genera in the seminal plasma of patients with moderate‐to‐severe AZS that displayed significant correlations with the metabolites, as depicted in Figure 6G. Among these genera, *Pseudomonas, Serratia*, and *Methylobacterium‐Methylorubrum* exhibited notable positive correlations with the core DEMs. Conversely, *Uruburuella, Vibrio*, and *Pseudoalteromonas* demonstrated significant negative correlations. These findings offer valuable insights for future research endeavors.

**Figure 7:**
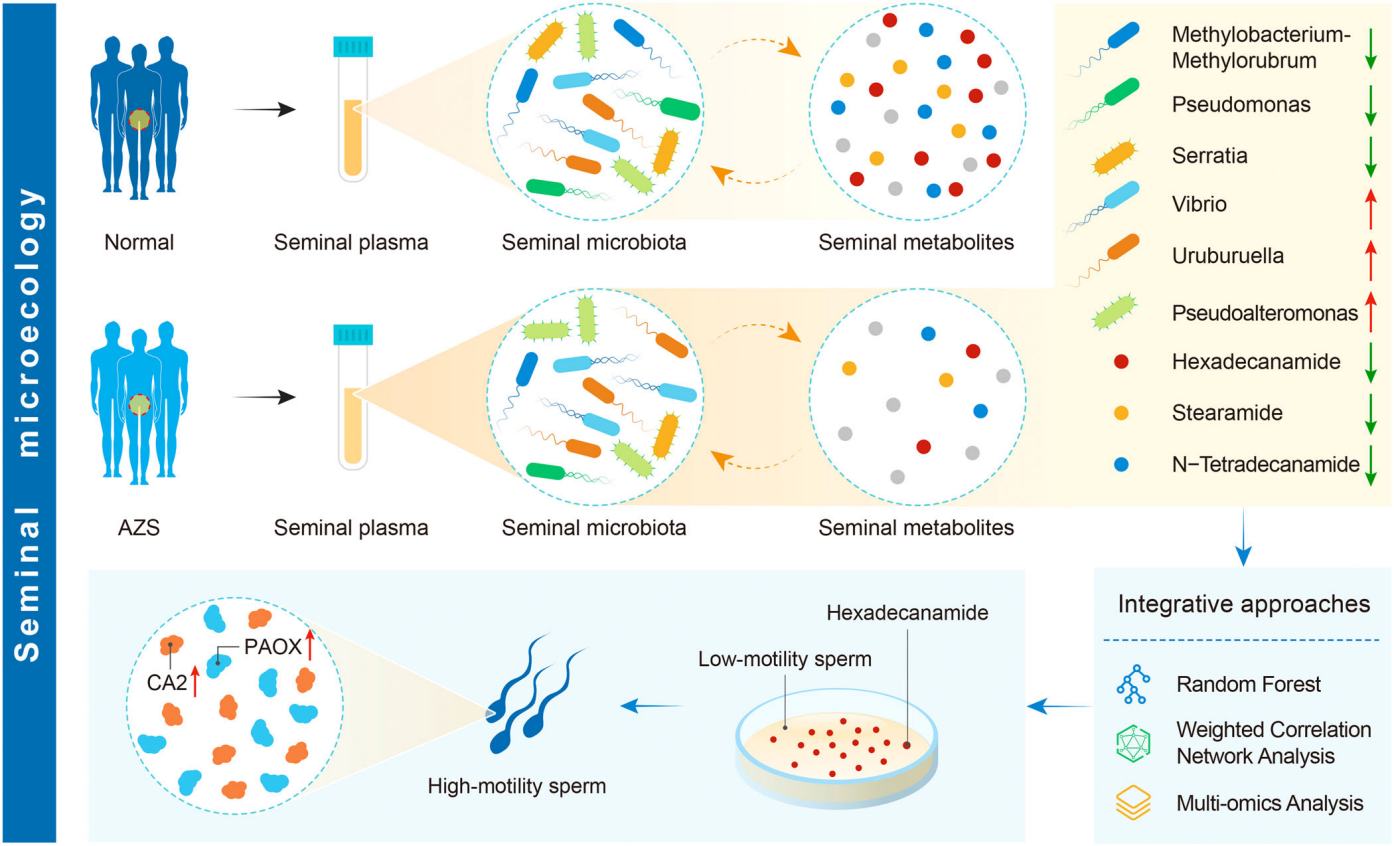
Conceptual map: Comprehensive overview of the biological process and methodological framework in study design. Patients diagnosed with AZS, the levels of three metabolites in seminal plasma are significantly reduced (n‐tetradecanamide, hexadecanamide, and stearamide). Through the integration of Random Forest, weighted gene co‐expression network analysis (WGCNA), and multi‐omics analysis, it was observed that the levels of six microbial genera (*Pseudomonas, Serratia, Methylobacterium‐Methylorubrum, Uruburuella, Vibrio*, and *Pseudoalteromonas*) in seminal plasma are significantly correlated with the concentration of hexadecanamide. Specifically, the abundance of *Pseudomonas, Serratia*, and *Methylobacterium‐Methylorubrum* is significantly downregulated, whereas the abundance of *Uruburuella, Vibrio*, and *Pseudoalteromonas* is significantly upregulated. Through in vitro experiments, an increase in hexadecanamide in seminal plasma was found to be associated with a significant increase in the protein levels of PAOX and CA2 proteins. This elevation in protein levels was concomitant with a marked enhancement in sperm motility.

Building upon the findings of our study, we propose the biological process illustrated in Figure 7. In this process, the six genera of bacteria (*Pseudomonas, Serratia, Methylobacterium‐Methylorubrum, Uruburuella, Vibrio*, and *Pseudoalteromonas*) present in the seminal plasma of AZS patients instigate dynamic changes that potentially inhibit the synthesis or increase the consumption of hexadecanamide. Consequently, a decrease in the hexadecanamide content in seminal plasma can lead to a subsequent decline in sperm motility. This reduction in motility is attributed to the downregulation of two key proteins, PAOX and CA2, within sperm cells.

## DISCUSSION

In this study, we conducted a comprehensive analysis of the dynamic changes in the microecology of seminal plasma in patients with AZS by employing a combination of 16 S rDNA sequencing and metabolomic analysis. Our primary objective was to uncover potential etiological factors contributing to the development of AZS. Notably, our investigation identified three specific metabolites, including hexadecanamide [15], and detected associations with six bacterial genera in seminal plasma that demonstrated significant correlations with the severity of AZS. Additionally, we established a noteworthy connection between the composition of these six bacterial genera in the seminal plasma of moderate‐to‐severe AZS patients and the levels of hexadecanamide.

A particularly groundbreaking discovery in our study is the positive influence of hexadecanamide on sperm motility. We confirmed this effect through a series of in vitro and in vivo experiments, marking the first report of hexadecanamide's potential to enhance sperm motility. Our findings suggest that hexadecanamide may achieve this effect by upregulating the protein levels of two specific proteins, PAOX and CA2 in sperm cells.

The crucial role of sperm motility in successful fertilization cannot be overstated [16]. An analysis of 1085 male infertility cases conducted in 2003 revealed a high prevalence of weak spermatozoa, with nearly 81.84% of male infertility patients experiencing reduced sperm motility [17]. These findings underscore the fundamental importance of sperm motility in male fertility. Recent research has increasingly emphasized the link between semen microbiota and AZS. Although previous studies have detected microbial communities in semen and identified specific microorganisms with potential impacts on sperm quality [18, 19, 20], a comprehensive understanding of the connection between these microbial communities and metabolomics has remained elusive. A key strength of our study lies in its use of multi‐omics approaches [21, 22] to investigate the microbiota and metabolome characteristics of seminal plasma. This comprehensive analysis allowed us to make more accurate assessments of the differences between AZS patients and healthy individuals.

Furthermore, our research uncovered significant associations between six bacterial genera, with a particular emphasis on *Pseudomonas*, and the development of moderate‐to‐severe AZS. Notably, our findings align with prior research indicating a negative correlation between the abundance of *Pseudomonas* and sperm concentration, while simultaneously noting a direct correlation with the total number of motile sperms [2]. However, it is essential to acknowledge the limitations of 16S rDNA sequencing technology [23], which restrict our ability to precisely identify the bacterial strains responsible for AZS. Nevertheless, this aspect remains a focal point for future research. It is of paramount importance to recognize that the six genera mentioned in this study comprise a multitude of individual strains, each characterized by distinct attributes and the potential to exert varying effects on sperm motility and male fertility. To propel our comprehension forward and enhance clinical approaches for diagnosing and treating AZS, forthcoming investigations will be devoted to the precise identification and validation of the specific strains within these genera that exhibit a robust association with impaired sperm motility. A more exhaustive exploration into the specific strains linked to AZS will serve to establish a finer‐grained and more exact correlation between microbial composition and male reproductive well‐being. This, in turn, will pave the way for more accurate diagnostic methodologies and targeted therapeutic interventions. An additional limitation of our study is the inability to utilize specific antibiotics to target and eliminate particular bacterial genera and thereby validate their pivotal roles in the development of moderate‐to‐severe AZS. This avenue presents a promising direction for future investigations.

In summary, our study highlights the significance of alterations in the microecological dynamics of seminal plasma as potential indicators or predictors of semen health. Future research could focus on identifying specific metabolites with abnormal elevations that may contribute to reduced sperm motility, and further assessing the impact of bacterial genera with atypical abundances on sperm motility. Undoubtedly, the elucidation of the microecological dynamics of seminal plasma is essential for gaining valuable insights into the health status of the male urogenital system.

## CONCLUSION

To the best of our knowledge, our study marks the pioneering evidence supporting the concept that hexadecanamide in seminal plasma may have the capacity to enhance sperm motility by increasing the protein levels of PAOX and CA2 proteins. Additionally, we uncovered a significant correlation between reduced hexadecanamide levels and the perturbations in six bacterial genera in seminal plasma, specifically in the context of moderate‐to‐severe AZS. These discoveries hold the potential for meaningful clinical applications, providing valuable insights into the management of AZS. In particular, they have the potential to inform diagnostic and therapeutic strategies for AZS and offer a robust theoretical foundation for the advancement of its clinical management.

## METHODS

The comprehensive methodology, encompassing experimental procedures, sequencing protocols, data processing techniques for sequencing data, and approaches for data analysis is available in Supplementary Information.

## AUTHOR CONTRIBUTIONS

Baoquan Han and Yongyong Wang provided the idea, designed the methodology, did the experiments, analyzed data, and wrote the manuscript. Wei Ge and Junjie Wang did the experiments. Shuai Yu, Jiamao Yan, Lei Hua, Xiaoyuan Zhang, Zihui Yan, Lu Wang, and Jinxin Zhao designed the methodology. Yunqi Zhang, Shaolin Liang, Jianjuan Zhao, Zhongyi Sun, Wei Shen, and Yaoting Gui revised the manuscript. Baoquan Han, Bo Yang, Zhongyi Sun, Wei Shen, and Yaoting Gui acquired financial support for the project. Cong Huang, Bo Yang, Yan Wang, and Qian Ma provided study materials, reagents, materials, laboratory samples, animals, instrumentation, computing resources, and other analysis tools. Yong Zhao and Hui Jiang supervised this project. All authors have read the final manuscript and approved it for publication.

## CONFLICT OF INTEREST STATEMENT

The authors have declared no competing interests.

## ETHICS STATEMENT

The ethics applications were approved by the Ethics Committee of Qingdao Municipal Hospital (No: SZYXLL‐2‐2021‐2‐002) and Committee on Laboratory Animal Welfare of Shenzhen‐Peking University‐The Hong Kong University of Science and Technology Medical Center (No: 2021‐850), respectively. Written informed consent was acquired from all individuals who participated in the research.

## Supporting information


**Figure S1**: AZS‐associated microbiota community structure and their species difference analysis.
**Figure S2**: Distribution of selected genera in different sample groups.
**Figure S3**: Box plots: abundance distribution of six core differential genera between sample groups.
**Figure S4**: AZS‐associated broad changes in the seminal plasma metabolic profile of diverse groups.
**Figure S5**: AZS‐associated broad changes in the seminal plasma metabolic profile of diverse groups.
**Figure S6**: Correlation analysis of metabolites across different sample groups.
**Figure S7**: KEGG enrichment analysis of differential metabolites in four comparison groups.
**Figure S8**: Preliminary study on the mechanism of hexadecanamide enhancing sperm motility in vitro and in vivo.
**Figure S9**: Expression analysis of *PAOX* and *CA2* genes in testicular tissue with single‐cell resolution.
**Figure S10**: AZS‐associated metabolic features and their microbial associations in different comparison groups.
**Figure S11**: Correlation plot of AZS‐associated metabolic features and their microbial of AZS‐II versus AZS‐I.

**Table S1**: Relevant patient and sample information.
**Table S2**: The metadata file that contains the corresponding ID of sample names aligned with the GSA identifier (CRA009103) as referenced in this study.
**Table S3**: Patient baseline characteristics.
**Table S4**: Information of standard‐labeled total metabolites and differential metabolites based on different databases.

## Data Availability

Metagenomic sequences for the Discovery and Validation cohorts are accessible through the Genome Sequence Archive at the BIG Data Center, Beijing Institute of Genomics, Chinese Academy of Sciences, under the identifier GSA CRA009103 (https://ngdc.cncb.ac.cn/gsa/browse/CRA009103). The metabolomics resources and data used in this study are accessible via the web at https://www.ebi.ac.uk/metabolights, cataloged with the accession number MTBLS7586. The new experimental data generated in this study is accessible for download on the following website: https://github.com/Baoquan-Han/AZS_Project/releases/download/v1.0.2/TableS.xlsx. Supporting Information (methods, figures, tables, scripts, graphical abstract, slides, videos, Chinese translated version and update materials) may be found in the online DOI or iMeta Science http://www.imeta.science/.
