# Supplementary material for: Changes in seminal plasma microecological dynamics and the mechanistic impact of core metabolite hexadecanamide in asthenozoospermia patients
Source: Imeta. 2024 Jan 25;3(2):e166. doi: 10.1002/imt2.166 (PMC11170967; doi:10.1002/imt2.166)
Supplement: Supplementary file 1 — Figure S1: AZS‐associated microbiota community structure and their species difference analysis. Figure S2: Distribution of selected genera in different sample groups. Figure S3: Box plots: abundance distribution of six core differential genera between sample groups. Figure S4: AZS‐associated broad changes in the seminal plasma metabolic profile of diverse groups. Figure S5: AZS‐associated broad changes in the seminal plasma metabolic profile of diverse groups. Figure S6: Correlation analysis of metabolites across different sample groups. Figure S7: KEGG enrichment analysis of differential metabolites in four comparison groups. Figure S8: Preliminary study on the mechanism of hexadecanamide enhancing sperm motility in vitro and in vivo. Figure S9: Expression analysis of PAOX and CA2 genes in testicular tissue with single‐cell resolution. Figure S10: AZS‐associated metabolic features and their microbial associations in different comparison groups. Figure S11: Correlation plot of AZS‐associated metabolic features and their microbial of AZS‐II versus AZS‐I. [file IMT2-3-e166-s001.docx]

Supporting information to:

**Changes in seminal plasma microecological dynamics and the mechanistic impact of the** **core metabolite hexadecanamide in asthenozoospermia patients**

**Running title**: The correlation between alterations in seminal microbiota and the onset of asthenozoospermia

Baoquan Han^1,2#^, Yongyong Wang^3#^, Wei Ge^4^, Junjie Wang^4^, Shuai Yu^2^, Jiamao Yan^4^, Lei Hua^1^, Xiaoyuan Zhang^4^, Zihui Yan^4^, Lu Wang^4^, Jinxin Zhao^4^, Cong Huang^5^, Bo Yang^2^, Yan Wang^6^, Qian Ma^2^, Yong Zhao^7^, Hui Jiang^2^, Yunqi Zhang^8^, Shaolin Liang^8,9^, Jianjuan Zhao^8^, Zhongyi Sun^1^*, Wei Shen^4^*, Yaoting Gui^2^*

^1^Department of Urology, Shenzhen University General Hospital, Shenzhen, China.

^2^Shenzhen Key Laboratory of Male Reproductive Medicine and Genetics, Institute of Urology, Peking University Shenzhen Hospital, Shenzhen-Peking University-The Hong Kong University of Science and Technology Medical Center, Shenzhen, China.

^3^Department of Reproductive Medicine, Qingdao Hospital, University of Healthy and Rehabilitation Sciences (Qingdao Municipal Hospital), Qingdao, China

^4^College of Life Sciences, Qingdao Agricultural University, Qingdao, China.

^5^Department of Dermatology, Skin Research Institute of Peking University Shenzhen Hospital, Peking University Shenzhen Hospital, Shenzhen Peking University-The Hong Kong University of Science and Technology Medical Center, Shenzhen, China.

^6^Department of Urology, Peking University Shenzhen Hospital, Shenzhen, China.

^7^State Key Laboratory of Animal Nutrition, Institute of Animal Sciences, Chinese Academy of Agricultural Sciences, Beijing, China.

^8^STI-Zhilian Research Institute for Innovation and Digital Health, Beijing, China.

^9^Institute for Six-sector Economy, Fudan University, Shanghai, China.

^#^These authors contributed equally: Baoquan Han, Yongyong Wang

*Correspondence: [zhy.sun@szu.edu.cn](mailto:zhy.sun@szu.edu.cn) (Zhongyi Sun), [wshen@qau.edu.cn](mailto:wshen@qau.edu.cn) (Wei Shen), [gyt@pkuszh.com](mailto:gyt@pkuszh.com) (Yaiting Gui)

The online version contains methods, supplementary figures and tables available.

**METHODS**

**Study design and inclusion/exclusion criteria**

This study’s cohort comprised patients diagnosed with asthenozoospermia (AZS) [1,2] at Qingdao Municipal Hospital (QMH, Qingdao, China). A total of 120 adult patients (*n* = 120), aged between 22 and 45 years, were included in the study. The cohort was divided into four groups based on the severity of AZS: mild AZS (AZS-I, *n =* 30), moderate AZS (AZS-II, *n =* 30), severe AZS (AZS-III, *n =* 30), and a control group with Normal semen parameters (Normal, *n =* 30). The diagnosis of AZS was made in accordance with the World Health Organization (WHO) laboratory manual for the examination and processing of human semen [3] (Figure 1A). Furthermore, we apply stringent criteria for the exclusion of non-compliant seminal specimens. Such exclusions encompass samples exhibiting a semen volume of less than 1mL and an abstinence duration falling below two days or exceeding seven days. Additionally, in instances where collected specimens display contamination by extraneous matter or are identified as hematospermia, their exclusion is imperative to safeguard the overall objectivity and reliability of the experimental outcomes. The research protocol was subjected to review and approval by the Institutional Review Board of Qingdao Municipal Hospital (SZYXLL-2-2021-2-002) and Committee on Laboratory Animal Welfare of Shenzhen-Peking University-The Hong Kong University of Science and Technology Medical Center (2021-850).

Semen samples were meticulously collected from eligible patients by the Department of Reproductive Medicine [4]. These samples were stored at -80°C until DNA extraction. It is noteworthy that to eliminate the risk of contamination from extraneous microbial sources within the laboratory environment, UV light disinfection was carried out one day prior to both sample collection and microbial nucleic acid extraction. Freshly collected semen samples subsequently accepted a series of routine semen clinical assessments, including computer-assisted semen analysis (CASA) system (Suiplus Semen Analysis-II, Beijing Suijia Software, China) and Gram staining [3]. Relevant patient and sample information can be found in Table S1 and Table S2.

Subsequent to semen collection and initial testing, the specimens were subjected to a specific protocol adhering to the guidelines outlined by the Department of Reproductive Medicine at Qingdao Municipal Hospital. The semen samples were meticulously processed to isolate sperm from seminal plasma through the implementation of the density gradient method. These solutions were subsequently allowed to equilibrate overnight in a preheated incubator. Approximately 4 ml of the solution was prepared for each patient. To facilitate the separation, density gradient centrifugation solutions (K-SISG-50, William A. Cook Australia, Bloomington) with concentrations of 80% and 40% were meticulously prepared. The procedure was initiated by introducing 1.5 mL of the 80% density gradient centrifugation solution to the base of a 5 ml tube, followed by the gentle layering of an equivalent volume of the 40% density gradient centrifugation solution atop it. This method resulted in the creation of a distinct and transparent interface within the tube. The freshly collected semen was then left to take liquefaction at room temperature for a period of 15-20 minutes. The liquefied semen was subsequently gently aspirated into the prepared gradient solution using a pasteur pipette. Following this step, the tubes were subjected to centrifugation at 400g for a duration of 15 minutes. Upon completion of centrifugation, the seminal plasma and the density gradient solution were extracted from the upper portion of the centrifuge tube and segregated into two distinct parts. One part was allocated for the subsequent 16S rDNA sequencing analysis, while the other part was designated for metabolomics analysis, which employed Ultra High-Performance Liquid Chromatography with Tandem Mass Spectrometry (UHPLC-MS/MS) methods.

The validation cohort was being prepared for future investigations. The validation cohort was constituted of 60 patients (*n =* 60), with an equal distribution of 30 individuals classified as Normal and 30 diagnosed with AZS, further subcategorized into 23 with AZS-II and 7 with AZS-III (Figure 1A). Semen samples from participants in the validation cohort were procured in strict accordance with the previously detailed protocol. Post-collection, these samples were meticulously preserved within a -80°C environment and remained in a frozen state until they took either protein or RNA extraction or *in vitro* cultivation for subsequent analysis. In order to validate the presence of microorganisms in our samples and exclude the possibility of contamination during laboratory procedures, we incorporated a negative control. This control is comprised of a sample of water and an equivalent blank matrix that is expected to be devoid of any microbial DNA.

**DNA extraction and library construction for 16S rDNA sequencing**

16S rDNA sequencing provides valuable insights into the diversity of genera and species within the seminal microbiota. It allows for the characterization of the microbial community, including the proportions of beneficial, pathogenic, and other microorganisms. In this section, the genomic DNA of the samples was extracted using the cetyltrimethylammonium bromide and sodium dodecyl sulfate (CTAB/SDS) method, and subsequently, the 16S rRNA genes spanning different regions (V3-V4) were amplified using specific primers (341F: CCTACGGGRBGCASCAG; 806R: GGACTACNNGGGTATCTAAT) accompanied by unique barcodes. The resulting Polymerase Chain Reaction (PCR) products took purification through the employment of the Qiagen Gel Extraction Kit (28704, Qiagen, Germany). Subsequently, sequencing libraries were meticulously prepared following the guidelines outlined in the NEBNext®Ultra™II DNA Library Preparation Kit (E7645, New England Biolabs, Massachusetts). The quality of the libraries was rigorously assessed employing the Qubit@2.0 Fluorometer (Thermo Fisher, Massachusetts) and the Agilent Bioanalyzer 2100 system. Ultimately, the libraries were subjected to sequencing on the Illumina NovaSeq platform (Illumina, California), generating 250 bp paired-end reads.

**Quality control and analysis of 16S rDNA sequencing data**

Quality control in 16S rDNA sequencing constitutes a pivotal stage that underpins dependable and reproducible research in the realm of microbiome and microbial community analysis. Raw sequence data is processed to remove low-quality sequences and adapter sequences. In the initial data processing, a series of bioinformatics tools were applied, including FLASH (v. 1.2.11), fastp (v. 0.20.0), and Vsearch (v. 2.15.0), for specific tasks such as merging the paired-end reads, quality filtering, and clean tags comparison. Subsequently, the denoising process was performed using the DADA2 module in the QIIME2 software (v. QIIME2-202006) to obtain the initial Amplicon Sequence Variants (ASVs), followed by the filtration of ASVs with abundances below 5. Taxonomic annotation was executed by leveraging the Silva database (v. 138.1) within the QIIME2 software.

For the assessment of diversity within the microbial communities, alpha diversity was quantified through seven distinct metrics, including Observed, Chao1, Shannon, Simpson, Dominance, Good’s coverage, and Pielou_e, all of which were computed using QIIME2. In addition, beta diversity was calculated based on the weighted and unweighted unifrac distances, providing insight into the differentiation between microbial communities. Cluster analysis was carried out employing principal component analysis (PCA), and the original variables were dimensionally reduced utilizing the ade4 package and the ggplot2 package in R software (v. 3.5.3). Principal coordinate analysis (PCoA) was employed to visualize disparities between various groups in multidimensional data. Furthermore, linear discriminant analysis effect size (LEfSe) analysis (with an LDA score threshold of 4) was performed using LEfSe software (v. 1.0) to identify potential biomarkers specific to AZS. Subsequently, functional annotation analysis was conducted using PICRUSt2 software (v. 2.1.2-b) to investigate the functional distinctions among microbial communities in different groups.

**Metabolite extraction and UHPLC-MS/MS analysis**

Seminal plasma specimens (100μL) were meticulously preserved in EP tubes. These specimens were subsequently subjected to resuspension by vortex mixing in 80% pre-cooled methanol. Following this preparation, the samples were injected into an LC-MS/MS system, employing well-established standard protocols for analysis. UHPLC-MS/MS analyses were conducted using a Vanquish UHPLC system (Thermo Fisher Scientific, Massachusetts) coupled with an Orbitrap Q ExactiveTM HF mass spectrometer (Thermo Fisher Scientific, Massachusetts) at Novogene Co., Ltd. (Beijing, China). Samples were injected onto a HypesilGold column (100×2.1 mm, 1.9μm) employing a 17-minute linear gradient at a flow rate of 0.2 mL/min. For positive polarity mode, eluent A (0.1% formic acid in water) and eluent B (methanol) were used, while for negative polarity mode, eluent A consisted of 5 mM ammonium acetate at pH 9.0, and eluent B was methanol. The solvent gradient was programmed as follows: 2% B for 1.5 min; 2-85% B over 3 min; 100% B for 10 min; 100-2% B over 10.1 min; 2% B for 12 min. The Q ExactiveTM HF mass spectrometer was operated in positive/negative polarity mode with a spray voltage of 3.5 kV, capillary temperature of 320°C, sheath gas flow rate of 35 arb, aux gas flow rate of 10 arb, S-lens RF level of 60, and aux gas heater temperature of 350°C.

**Metabolite profiling of semen samples**

The initial raw data generated by the UPLC-MS/MS were processing within Compound Discoverer 3.1. (Thermo Fisher Scientific, Massachusetts). This processing including the steps of peak comparison, peak selection, and individual metabolite quantification. Subsequent statistical analyses were executed using a combination of statistical software packages, R (v. 3.4.3), Python (v. 2.7.6), and CentOS (v. 6.6). Metabolite annotation was achieved through reference to multiple databases, including Kyoto Encyclopedia of Genes and Genomes (KEGG) (<https://www.genome.jp/kegg/>), Human Metabolome Database (HMDB) (<https://hmdb.ca/>) and Lipid Maps Database (<https://www.lipidmaps.org/>). PCA and partial least squares discriminant analysis (PLS-DA) were conducted using the metaX software. Differential metabolites were identified based on specific criteria: A variable importance in projection (VIP) score exceeding 1, a *p*-value less than 0.05, and a fold change (FC) of either greater than or equal to 2, or less than or equal to 0.5. Volcano plots were generated using ggplot2 to visually represent the identified differential metabolites. To delve further into their relationships, the correlation between these metabolites was examined utilizing the cor.mtest() function with Pearson’s method in R. Statistical significance was established at a threshold of *p*-values less than 0.05, and correlation plots were constructed using the corrplot package in the R. Moreover, the functions and metabolic pathways associated with these identified metabolites were thoroughly investigated in accordance with the KEGG database.

**Correlation analysis between microbiomes and metabolite in semen of AZS patients**

The correlation analysis relied on the computation of Pearson’s correlation coefficients. To visually represent the relationships and degrees of association between species diversity and metabolites, heat maps were meticulously constructed. The correlation coefficients calculated for these associations ranged between -1 and 1. Notably, a correlation coefficient less than 0 indicated a negative correlation, whereas a coefficient greater than 0 signified a positive correlation. Equally, a correlation coefficient equal to 0 indicated a zero correlation, thus implying the absence of any discernible association between the variables under consideration.

**Random Forest classification analysis and differential metabolites analysis**

The microbial genomic data derived from 16S rDNA sequencing were used to establish predictive models aimed at identifying patients with AZS. By employing the random forests machine learning algorithm based on MicrobiomeAnalystR package [5], four distinct classification models were created. Each model was specifically designed to predict one of the following scenarios: (1) Normal/AZS-II state, (2) Normal/AZS-III state, (3) AZS-I state/AZS-II state and (4) AZS-I state/AZS-III state. The training dataset for these models consisted of patients from the discovery cohort. A total of 30 predictive variables were selected for model construction based on their variable importance (Figure 2A). We evaluated the models using ROC curves and conducted the analysis using the calcAUROC function within the R from the metaX package.

We also constructed ROC curves for differential metabolites. The ROC curve, also referred to as the receiver operating characteristic curve or sensitivity curve, is generated by plotting various binary classification methods (thresholds or decision domains). The ROC curve for differential metabolites serves as an assessment tool for potential biomarkers. On the graph, the horizontal axis represents the false positive rate (1-specificity), while the vertical axis represents the true positive rate (sensitivity). The curve was generated using the calcAUROC () function within the metaX package of R software.

**Weighted gene co-expression network analysis (WGCNA) of seminal plasma metabolites in AZS patients and screening of candidate metabolites**

We initiated our analysis by screening the core differentially metabolites (DEMs) through correlation analysis and multi-group intersection. Subsequently, we conducted WGCNA on the metabolite profiling using the WGCNA package [6−8], aimed at exposing intergroup correlations. DEMs (with *p*-values < 0.05 and VIP-values ≥ 1) were subjected to R software for the identification of high-quality metabolites and samples. To ensure the construction of a scale-free network, we set the power of β to 6. Hierarchical clustering dendrogram was employed to summarize the metabolite modules, represented by different colors. Heat maps and topological overlap matrix (TOM) diagrams were used for visualizing module structures. Leveraging WGCNA profiling, we intersected module-related DEMs with core DEMs to screen the most influential candidate metabolites.

**Quantitative analysis of diverse sample candidate metabolites**

The quantification of hexadecanamide was conducted using an Ultra High-Performance Liquid Chromatography (Ultimate 3000RS, Thermo Fisher Scientific, Massachusetts) coupled to a high-resolution Q-Orbitrap mass spectrometry system (Thermo Fisher Scientific, Massachusetts). Separation was carried out on a Welch Xtimate UHPLC C18 column (2.1×100mm, 1.8μm) maintained at 40°C. The mobile phase, comprising 0.1% formic acid in 5mM ammonium formate (solvent A) and 0.1% formic acid in acetonitrile (solvent B), was delivered at a flow rate of 0.30 mL/min. The solvent gradient was programmed as follows: 0-1min, 70%B; 1-9min, 70-98%B; 9-13min, 98%B; 13-13.5min, 98-70%B; 13.5-17.5min, 70%B. The mass spectrometer operated in alternate modes of Full MS Scan and Parallel Reaction Monitoring (PRM). Ion Source Parameters were set as follows: Spray Voltage (3.8kV); Sheath Gas (35 arb); Auxiliary gas (15 arb); Sweep gas (2psi); Auxiliary gas heater temperature 320℃; S-lens RF level 50V. In full-scan mode, the following parameters were employed: resolving power at 140,000FWHM; Scan range 100-500 m/z; Automatic Gain Control (AGC) target at 1×10^6^, maximum ion injection time 100ms. Fragmentation in PRM mode was achieved through high-energy collisional dissociation (HCD) at 80% normalized collision energy, with a resolving power of 70,000FWHM and AGC target at 2×10^5^.

**Target gene prediction and expression profiling in multiple databases for candidate metabolites**

Upon confirmation of the candidate metabolites, we employed the Swiss Target-Prediction webtool (<http://swisstargetprediction.ch/>) to predict the target genes associated with these candidate metabolites. Subsequently, the candidate genes took filtration and sorting based on their probability values. Following this, we analyzed the expression profiles of these target genes using various databases, including microarray data from the Gene Expression Omnibus (GEO) database (GSE6968 and GSE6872), along with testis-related single RNA-seq data accessible from The Human Protein Atlas (<https://www.proteinatlas.org/>).

***In vitro* co-culture of sperm cells with candidate metabolites and sperm motility assessment**

Following the identification of candidate metabolites and target genes, a preliminary *in vitro* experiment was conducted to validate the impact of the candidate metabolite and determine the optimal culture concentration. The experimental process proceeded as follows: semen specimens were classified according to the standard WHO criteria, and samples with sperm motility of less than 20% were categorized as the AZS-II or AZS-III group. These specimens were obtained through direct washings, and the sperm concentration was equally adjusted to create four separate portions. Subsequently, these four sperm samples were introduced into a sperm culture medium (510368, G-IVFTM PLUS, Vitrolife, Sweden) *in vitro* and were assigned varying concentrations of hexadecanamide (H0067, TCI, Japan): 0 nM, 1 nM, 10 nM, and 100 nM, with hexadecanamide dissolved in ethanol at the specified concentrations. The final ethanol concentration was less than 1‰. The final sperm concentration in each replicate was set at 5×10^6^ sperm/ml, and the samples were incubated at 37 °C. Sperm motility was subsequently statistically analyzed at different time points (0 h, 24 h, 48 h, and 72 h).

Upon the completion of the preliminary experiment, 30 Normal sperm samples and 30 AZS-II or AZS-III sperm samples were collected. The AZS-II or AZS-III specimens were obtained through direct washings, and the sperm concentration was equally adjusted into four portions. Simultaneously, semen samples were collected from fertile males with sperm motility exceeding 32%. High-quality sperm samples were prepared using density gradient centrifugation. The sperm cells were then cultured *in vitro* according to the concentrations determined in the preliminary experiment. Finally, sperm motility was assessed using CASA.

**Detection of candidate target protein levels in sperm cells**

Following the sperm motility assay, sperm samples from various treatment groups (a total of 60 cases) were collected and lysed using radio-immunoprecipitation assay (RIPA) cell lysis solution (P0013C, Beyotime, China). To further confirm the mechanism by which the metabolite promotes sperm motility, the level of target proteins in different treatment groups was compared and detected through standard Western blotting procedures [9]. In brief, sperm samples were combined and subsequently subjected to extraction with RIPA lysis buffer for 30 minutes on ice to extract total proteins. The target proteins were electrophoresed on a 4%-10% sodium dodecyl sulfate gel (S8010, Solarbio, China) and then transferred onto a PVDF membrane (ISEQ00010, MilliporeSigma, Massachusetts). Primary antibodies (Anti-PAOX Rabbit polyclonal IgG antibodies, ER65441, HUABIO, China; Anti-CA2 Rabbit polyclonal IgG antibodies, A18034, ABclonal, China) were incubated with the membranes overnight at 4°C, and secondary antibodies (HRP-coupled goat anti-rabbit IgG antibody, A0208, Beyotime, China) were incubated at room temperature for 1 hour. The membranes were then treated with a BeyoECL Plus kit (P0018, Beyotime, China) for chemiluminescence and imaged using a chemiluminescence (ECL) detection system (Tanon 5200, Tanon, China).

**Detection of the promoting effect of candidate metabolites on *in vitro* sperm viability**

In this experimental phase, we established an oligoasthenospermia mouse model using busulfan (20mg/kg) (B2635, MilliporeSigma, Massachusetts) by following established procedures outlined in past research [10−12]. Initially, 25mg of busulfan was dissolved in a 15ml centrifuge tube containing a mixture of 5ml of DMSO (D8370, Solarbio, China) and 5ml of physiological saline. After complete dissolution, the centrifuge tube was placed in warm tap water to maintain the solution’s temperature. We selected 3-week-old ICR male mice (Vital River Laboratory Animal Technology, China) as subjects and recorded their individual body weights. The mice were evenly distributed into different groups based on their body weight, and each mouse received an injection of 8μl/g (8μL for every 1g of body weight). Throughout the injection process, the tube containing the medication was kept in warm tap water to maintain its temperature.

The mice were divided into four groups: a control group (Control), a group receiving intraperitoneal injections of candidate metabolites (H), a group receiving busulfan (Busulfan), and a group receiving both busulfan and metabolites simultaneously (Busulfan + H). Each group consisted of six mice. The intraperitoneal injection concentration of the candidate metabolites was determined based on the concentration identified during the preliminary screening.

After one full spermatogenesis cycle, we collected testicular tissues from mice in different treatment groups for morphological examination and testicular coefficient analysis. We employed computer-assisted sperm analysis system (Sperm Class Analyzer® CASA System, MICROPTIC, Spain) to statistically evaluate sperm quality and motility in different treatment groups, thereby confirming the impact of metabolites on sperm vitality.

**Statistical analyses**

All data were presented as mean ± standard deviation (SD), and statistical analyses were performed using Student’s t-test or one-way analysis of variance (ANOVA). Data comparisons between groups were conducted using the Dunnett’s T3 method or the Fisher least significant difference (LSD) method. A significance level of *p*-value < 0.05 was considered statistically significant. Each experimental measurement was replicated at least three times.

**REFERENCES**

1. Heidary, Zohreh, Kioomars Saliminejad, Majid Zaki-Dizaji, Hamid Reza Khorram Khorshid. 2020. “Genetic aspects of idiopathic asthenozoospermia as a cause of male infertility.” *Human Fertility* 23: 83−92. <https://doi.org/10.1080/14647273.2018.1504325>

2. Tu, Chaofeng, Weili Wang, Tongyao Hu, Guangxiu Lu, Ge Lin, Yue-Qiu Tan. 2020. “Genetic underpinnings of asthenozoospermia.” *Best Practice & Research Clinical Endocrinology & Metabolism* 34: 101472. <https://doi.org/10.1016/j.beem.2020.101472>

3. World Health Organization. 2010. “WHO laboratory manual for the examination and processing of human semen.”

4. Hou, Dongsheng, Xia Zhou, Xue Zhong, Matthew L. Settles, Jessica Herring, Li Wang, Zaid Abdo, Larry J. Forney, Chen Xu. 2013. “Microbiota of the seminal fluid from healthy and infertile men.” *Fertility and Sterility* 100: 1261−1269.e1263. <https://doi.org/10.1016/j.fertnstert.2013.07.1991>

5. Chong, Jasmine, Peng Liu, Guangyan Zhou, Jianguo Xia. 2020. “Using MicrobiomeAnalyst for comprehensive statistical, functional, and meta-analysis of microbiome data.” *Nature Protocols* 15: 799−821. <https://doi.org/10.1038/s41596-019-0264-1>

6. Langfelder, Peter, Steve Horvath. 2008. “WGCNA: an R package for weighted correlation network analysis.” *BMC Bioinformatics* 9: 559. <https://doi.org/10.1186/1471-2105-9-559>

7. Han, Baoquan, Zihui Yan, Shuai Yu, Wei Ge, Yaqi Li, Yan Wang, Bo Yang, Wei Shen, Hui Jiang, Zhongyi Sun. 2021. “Infertility network and hub genes for nonobstructive azoospermia utilizing integrative analysis.” *Aging* 13: 7052−7066. <https://doi.org/10.18632/aging.202559>

8. Han, Baoquan, Lu Wang, Shuai Yu, Wei Ge, Yaqi Li, Hui Jiang, Wei Shen, Zhongyi Sun. 2021. “One potential biomarker for teratozoospermia identified by in-depth integrative analysis of multiple microarray data.” *Aging* 13: 10208−10224. <https://doi.org/10.18632/aging.202781>

9. Han, Baoquan, Kun Xu, Zhongtian Liu, Wei Ge, Simin Shao, Pengcheng Li, Nana Yan, Xinyi Li, Zhiying Zhang. 2019. “Oral yeast-based DNA vaccine confers effective protection from *Aeromonas hydrophila* infection on *Carassius auratus*.” *Fish & Shellfish Immunology* 84: 948−954. <https://doi.org/10.1016/j.fsi.2018.10.065>

10. Han, Baoquan, Jiachen Guo, Bo Zhou, Chunxiao Li, Tian Qiao, Lei Hua, Yinuo Jiang, *et al*. 2023. “Chestnut polysaccharide rescues the damaged spermatogenesis process of asthenozoospermia-model mice by upregulating the level of palmitic acid.” *Frontiers in Endocrinology* 14: 1222635. https://doi.org/10.3389/fendo.2023.1222635

11. Pengfei, Zhang, Feng Yanni, Li Lan, Ge Wei, Yu Shuai, Hao Yanan, Shen Wei, *et al*. 2021. “Improvement in sperm quality and spermatogenesis following faecal microbiota transplantation from alginate oligosaccharide dosed mice.” *Gut* 70: 222. <https://doi.org/10.1136/gutjnl-2020-320992>

12. Zhao, Yong, Pengfei Zhang, Wei Ge, Yanni Feng, Lan Li, Zhongyi Sun, Hongfu Zhang, Wei Shen. 2020. “Alginate oligosaccharides improve germ cell development and testicular microenvironment to rescue busulfan disrupted spermatogenesis.” *Theranostics* 10: 3308−3324. <https://doi.org/10.7150/thno.43189>


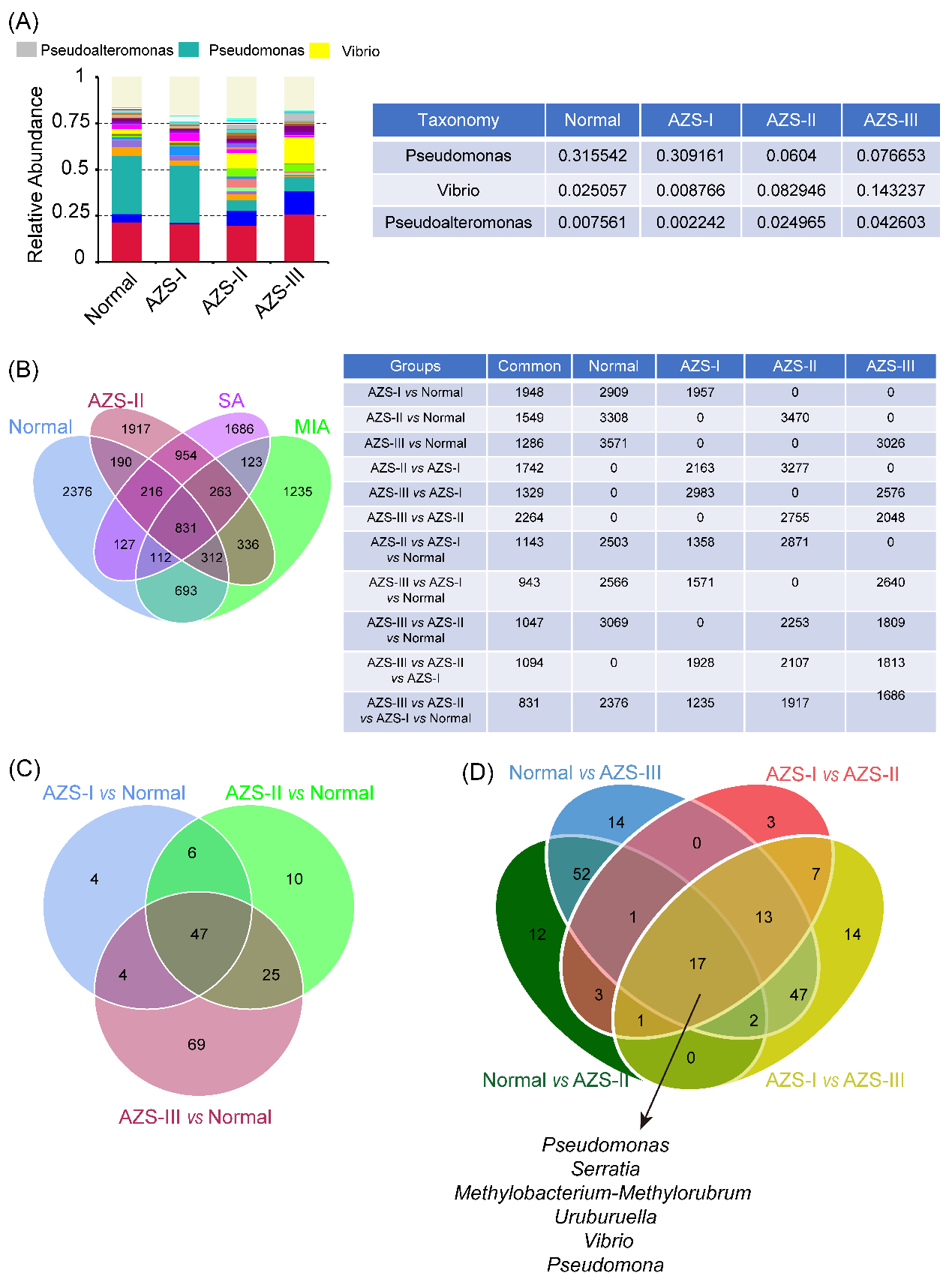


Figure S1 AZS-associated microbiota community structure and their species difference analysis. (A) Cumulative bar chart: top 30 species abundance in seminal plasma of AZS patients. (B) ASV analysis: seminal plasma microbiota in diverse AZS patients. (C) Analysis diagram: seminal plasma microbiota genera differences between diverse AZS patients and Normal donors. (D) Analysis diagram: seminal plasma microbiota genera differences in different comparison groups.

**
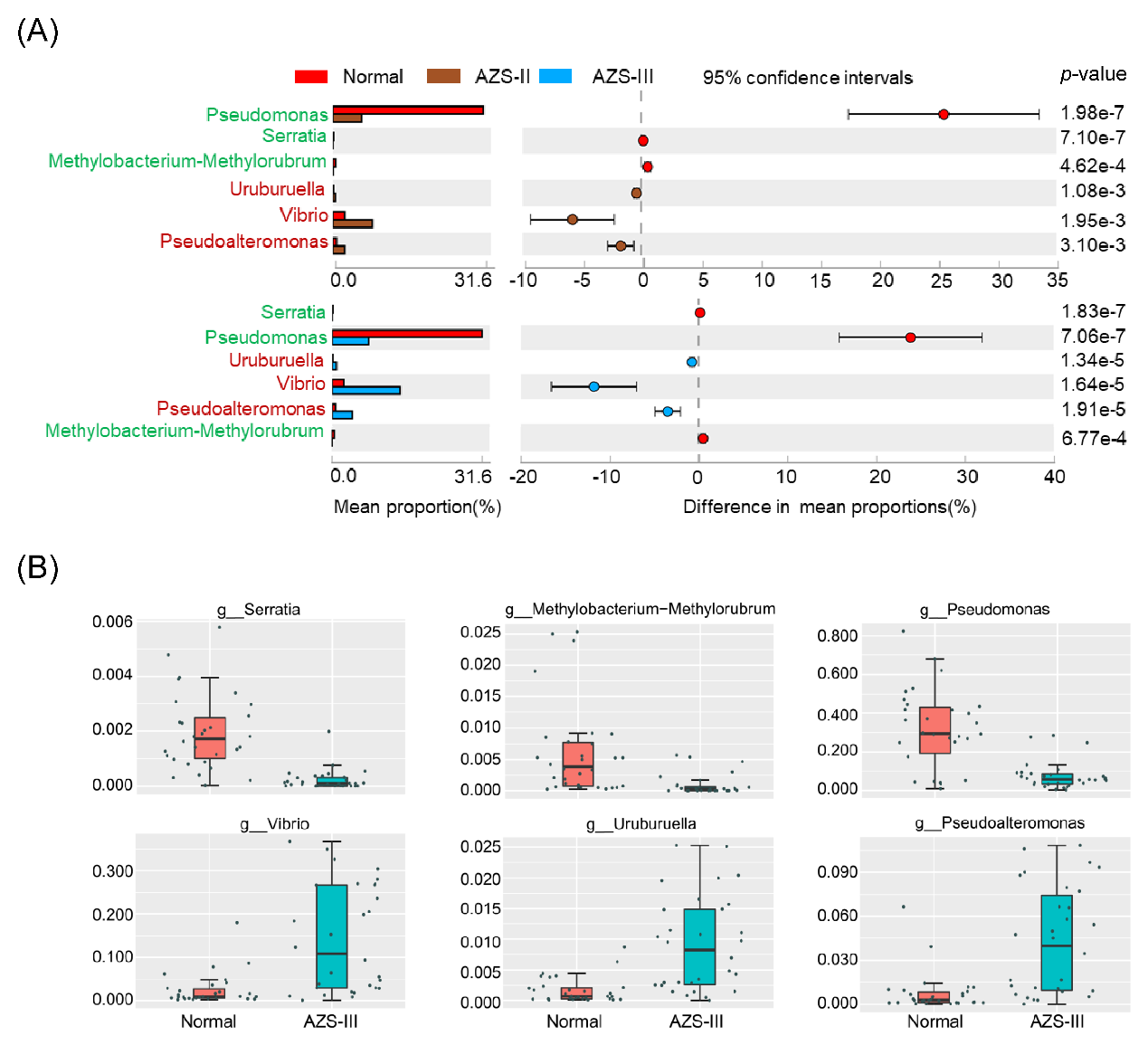
**

Figure S2 Distribution of selected genera in different sample groups. (A) STAMP analysis: species differences between sample groups for selected genera. (B) Box plots: abundance distribution of differential species among sample groups for selected genera.


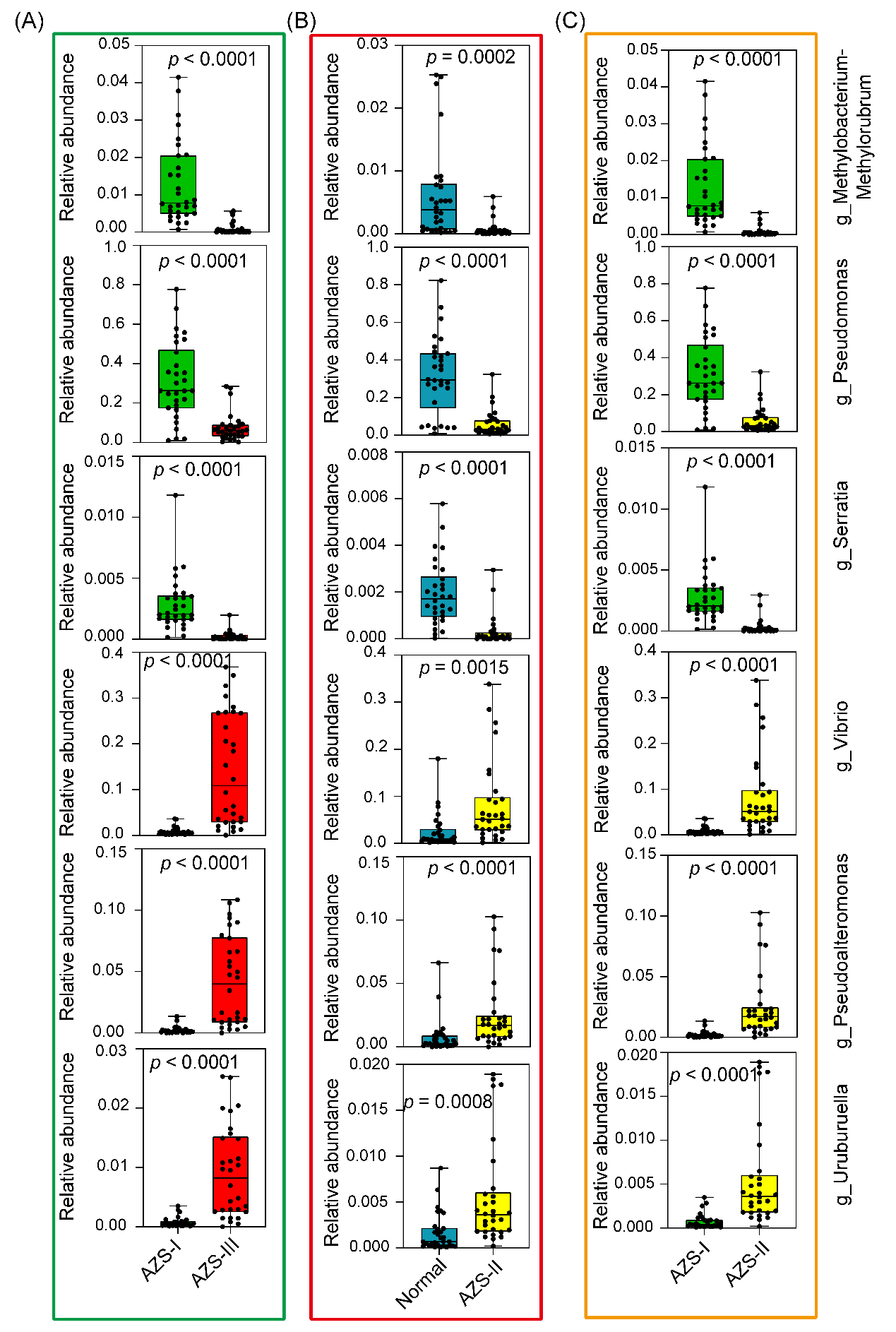


Figure S3 Box plots: abundance distribution of six core differential genera between sample groups. (A) abundance distribution of six core differential genera between AZS-I *vs* AZS-III. (B) abundance distribution of six core differential genera between Normal *vs* AZS-II. (C) abundance distribution of six core differential genera between AZS-I *vs* AZS-II. *p* < 0.05 indicates significant difference.

**
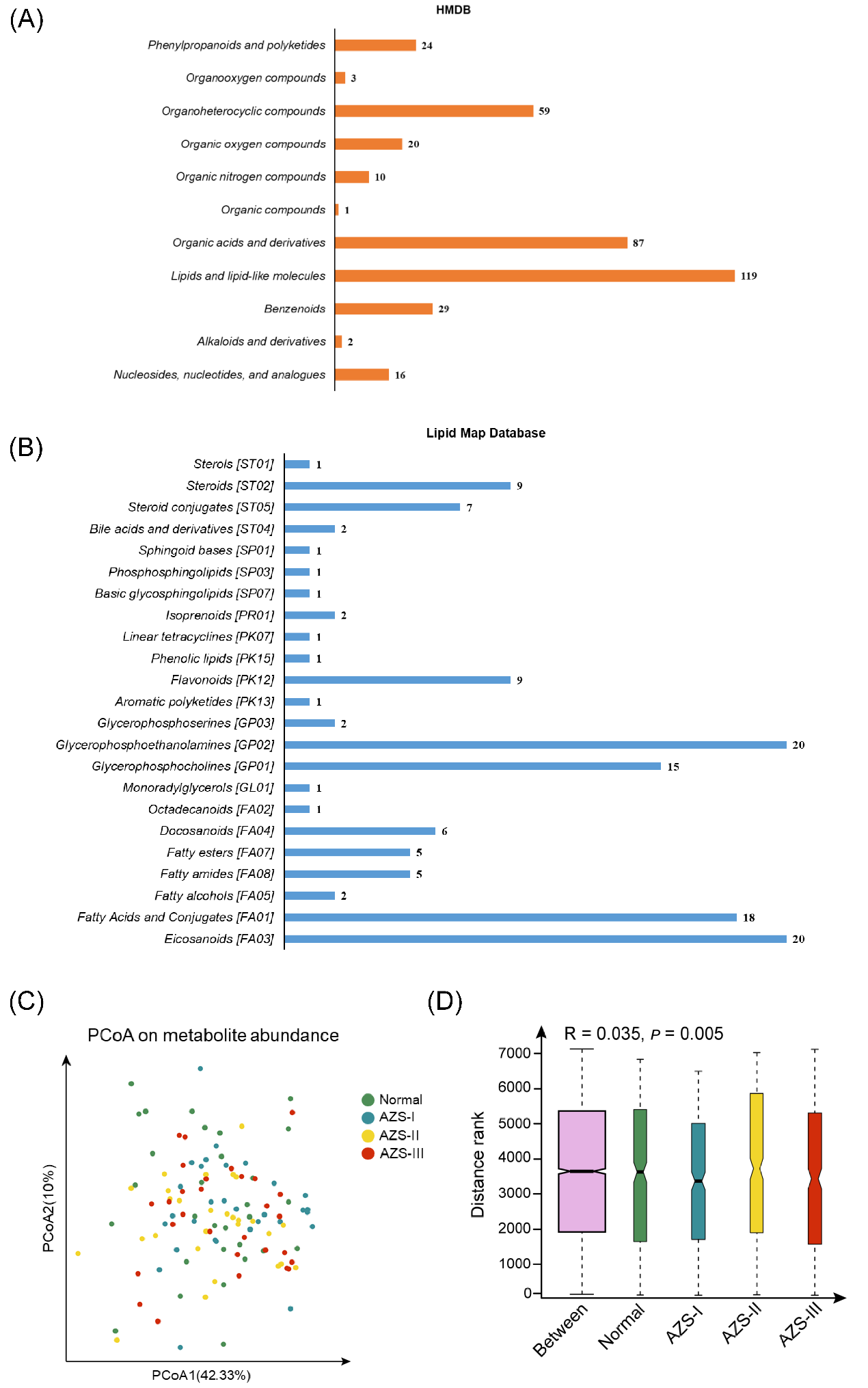
**

Figure S4 AZS-associated broad changes in the seminal plasma metabolic profile of diverse groups. (A) Metabolite distribution by metabolic classes (HDMB database) in AZS-associated changes. (B) Metabolite distribution by metabolic classes (Lipid Maps Database) in AZS-associated changes. (C) PCoA analysis: group differences in seminal plasma metabolic profiles. (D) Anosim analysis: differential metabolites across different sample groups.


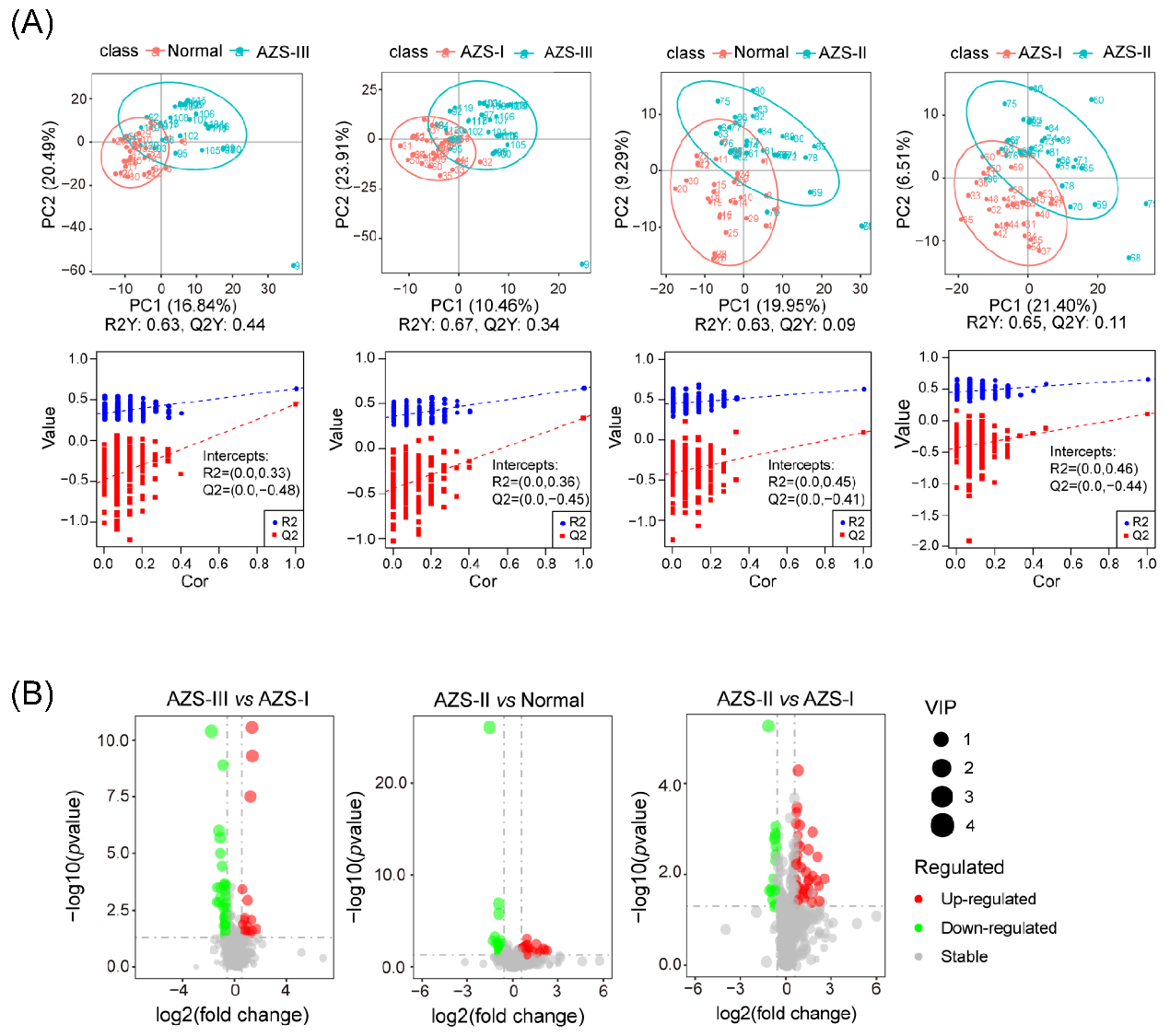


Figure S5 PLS-DA analysis and differential metabolites of the seminal plasma from diverse groups. (A) PLS-DA analysis: semen sample differentiation in AZS-associated changes among diverse groups. (B) Volcano map: differential metabolites in various comparison groups. Red dots represent Up-regulated metabolites, green dots represent Down-regulated metabolites, gray dots represent no change.

**
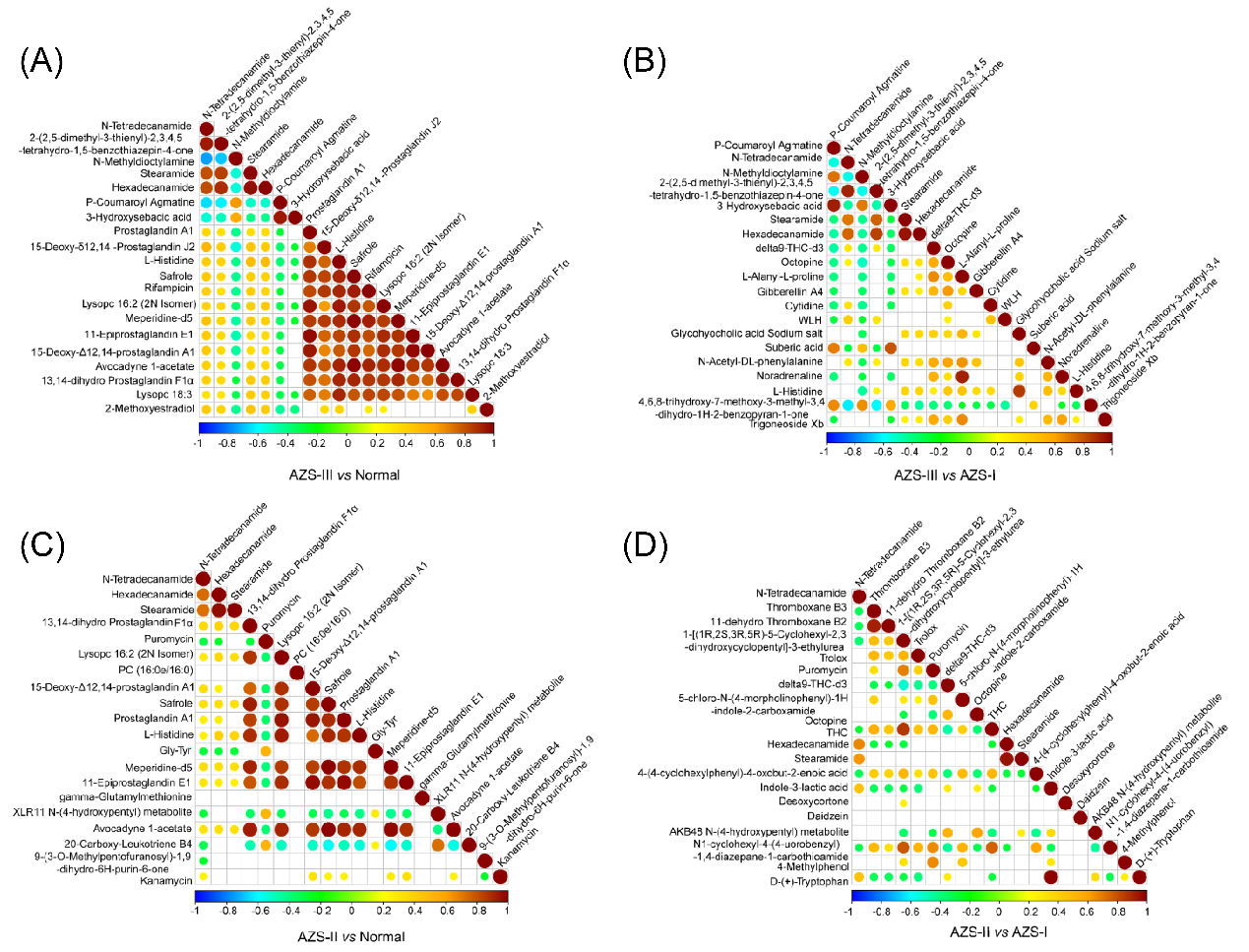
**

Figure S6 Correlation analysis of metabolites across different sample groups. (A) Correlation plot of metabolites of AZS-III *vs* Normal. (B) Correlation plot of metabolites of AZS-III *vs* AZS-I. (C) Correlation plot of metabolites of AZS-II *vs* Normal. (D) Correlation plot of metabolites of AZS-II *vs* AZS-I. The values in each cell of the plot are the correlation coefficients between the corresponding pairs of variables. The correlation coefficient ranges from -1 to 1. The size of the cells used to represent the magnitude of the correlation. Larger cells indicate stronger correlations.

**
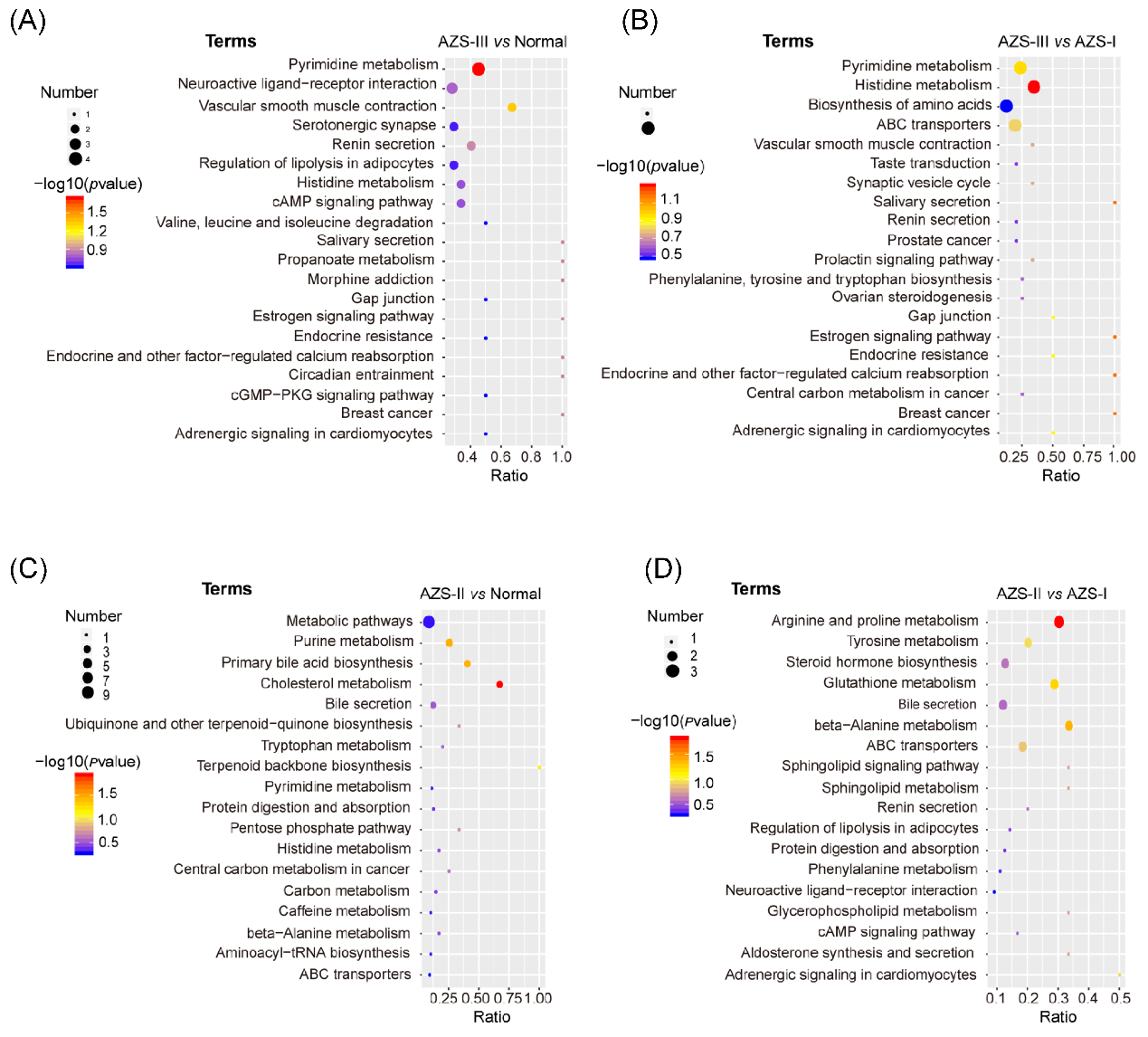
**

Figure S7 KEGG enrichment analysis of differential metabolites in four comparison groups. (A) KEGG enrichment plot of AZS-III *vs* Normal. (B) KEGG enrichment plot of AZS-III *vs* AZS-I. (C) KEGG enrichment plot of AZS-II *vs* Normal. (D) KEGG enrichment plot of AZS-II *vs* AZS-I. The plot displays individual pathways along the y-axis, and the significance levels (-log10 (*p*-value)) along the x-axis.


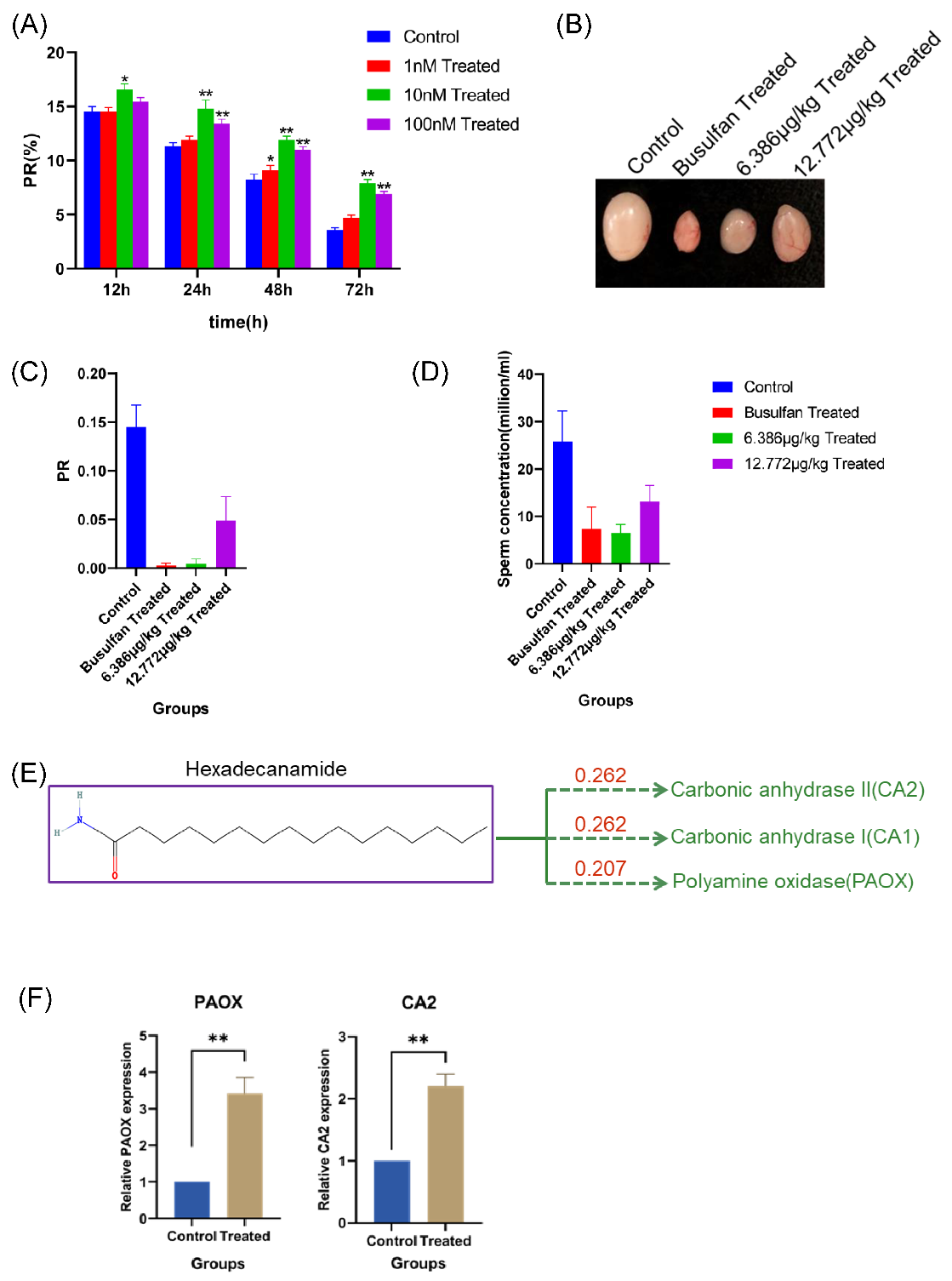


Figure S8 Preliminary study on the mechanism of hexadecanamide enhancing sperm motility *in vitro* and *in vivo*. (A) *In vitro* sperm motility observation in different treatment groups. (B) *In vivo* testes morphological observation in different treatment groups. (C) *In vivo* sperm motility observation in different treatment groups. (D) *In vivo* sperm concentration in different treatment groups. (E) Predicting hexadecanamide’s target genes using the Swiss-TargetPrediction database. (F) Protein level detection of PAOX and CA2 proteins in different *in vitro* treatment groups. *indicates *p*-value < 0.05, **indicates *p*-value < 0.01.


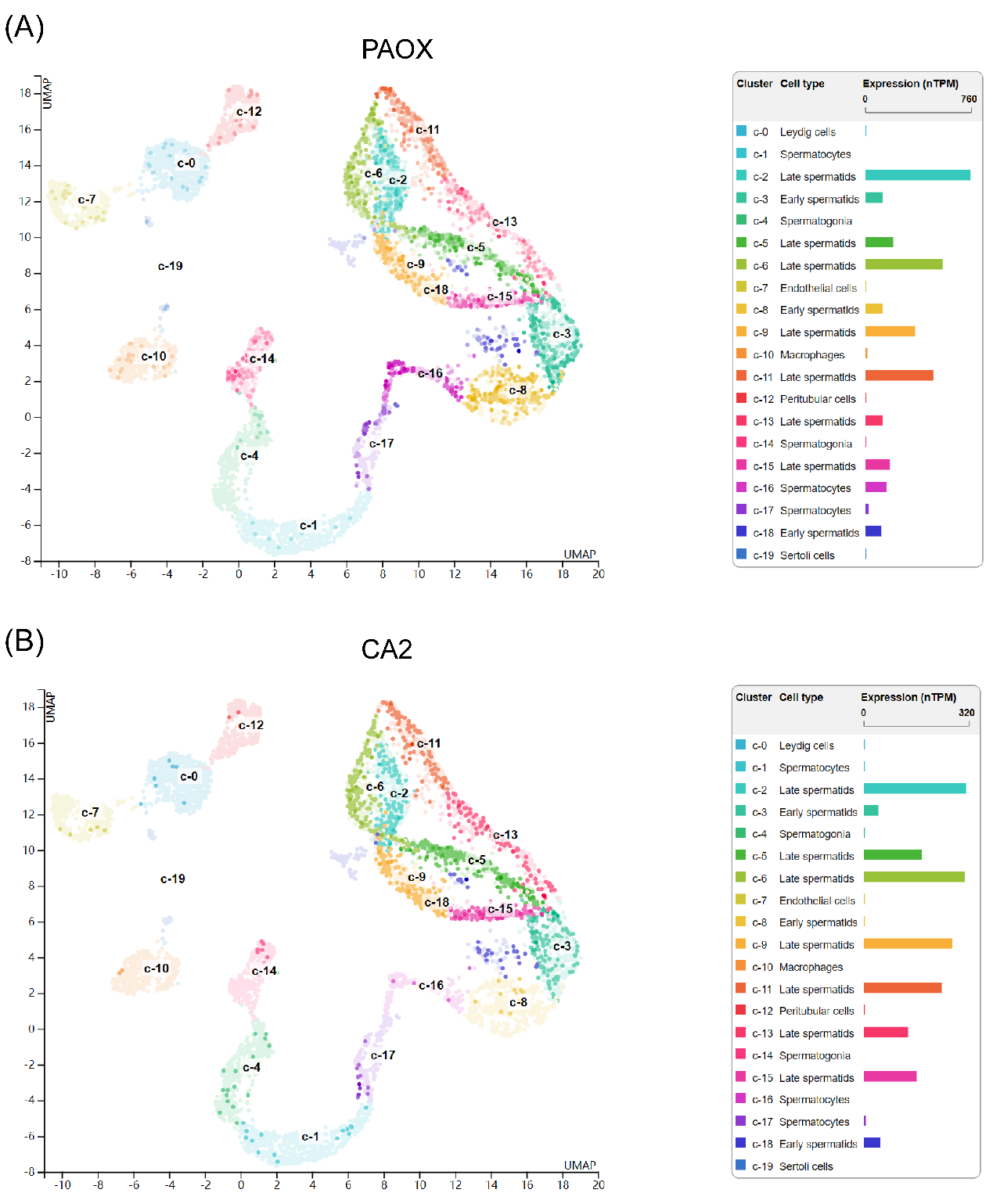


Figure S9 Expression analysis of *PAOX* and *CA2* genes in testicular tissue with single-cell resolution. (A). The expression levels of *PAOX* in different types of cells in the testis. (B) The expression levels of *CA2* in different types of cells in the testis.

**
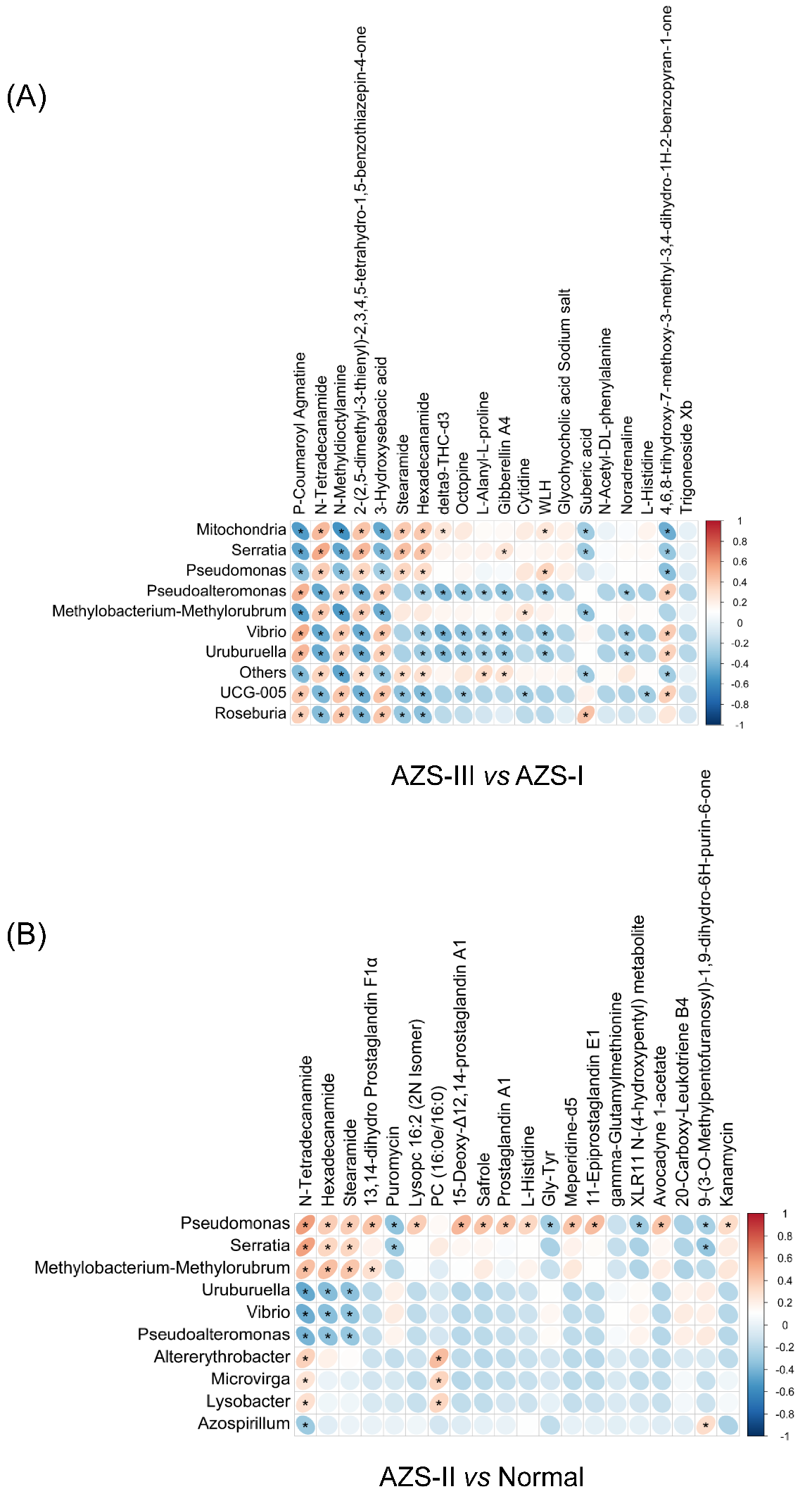
**

Figure S10 AZS-associated metabolic features and their microbial associations in different comparison groups. (A) Correlation plot of AZS-associated metabolic features and their microbial of AZS-III *vs* AZS-I. (B) Correlation plot of AZS-associated metabolic features and their microbial of AZS-II *vs* Normal. The values in each cell of the plot are the correlation coefficients between the corresponding pairs of variables. The correlation coefficient ranges from -1 to 1. The color of the cells used to represent the magnitude of the correlation. Darker color of cells indicates stronger correlations. * indicates significant difference (*p* < 0.05).

**
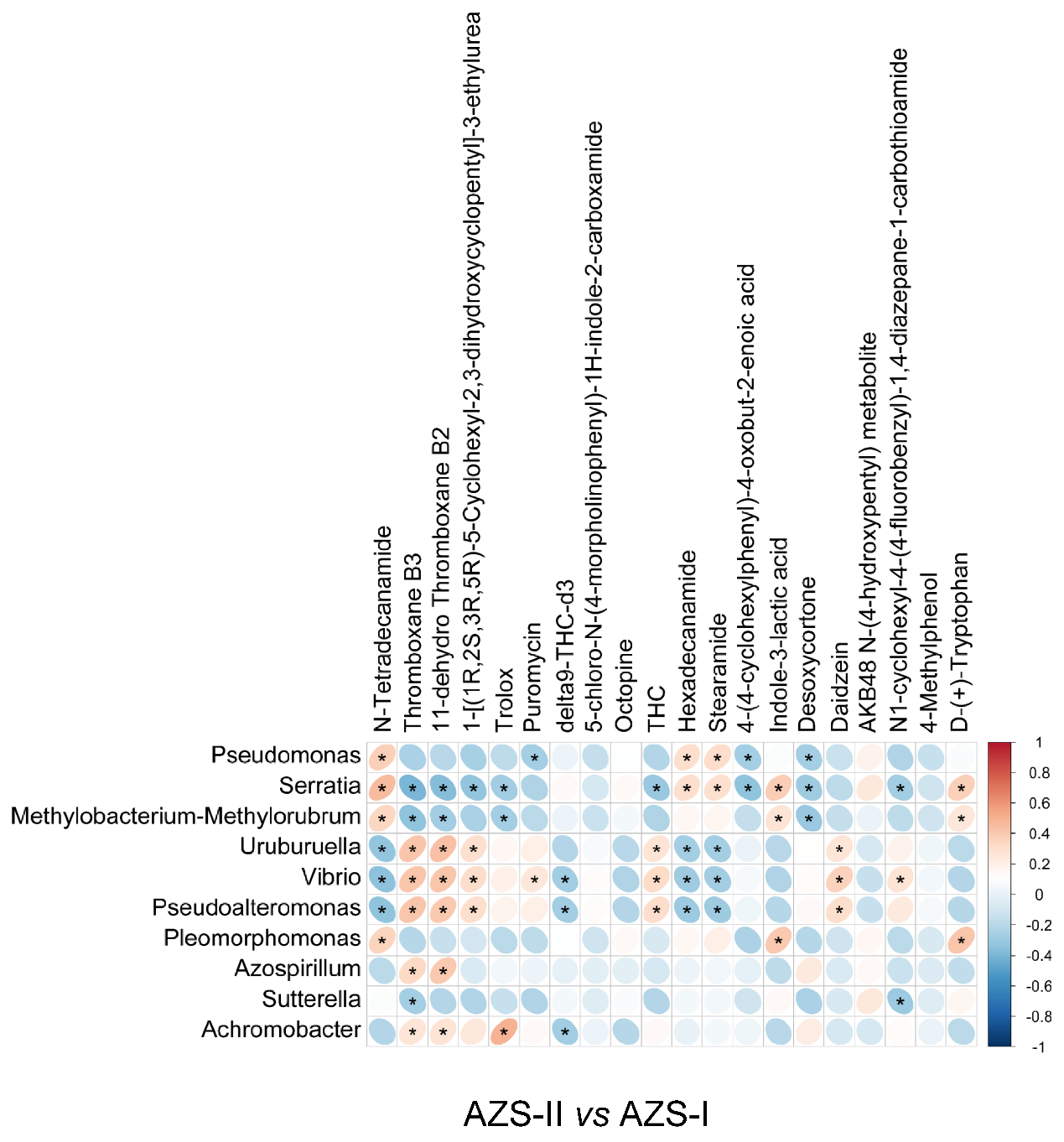
**

Figure S11 Correlation plot of AZS-associated metabolic features and their microbial of AZS-II *vs* AZS-I. The values in each cell of the plot are the correlation coefficients between the corresponding pairs of variables. The correlation coefficient ranges from -1 to 1. The color of the cells used to represent the magnitude of the correlation. Darker color of cells indicates stronger correlations. * indicates significant difference (*p* < 0.05).
